# Targeting Rab7‐Rilp Mediated Microlipophagy Alleviates Lipid Toxicity in Diabetic Cardiomyopathy

**DOI:** 10.1002/advs.202401676

**Published:** 2024-06-05

**Authors:** Jiahan Ke, Jianan Pan, Hao Lin, Shuying Huang, Junfeng Zhang, Changqian Wang, Alex Chia Yu Chang, Jun Gu

**Affiliations:** ^1^ Department of Cardiology Shanghai Ninth People's Hospital Shanghai Jiaotong University School of Medicine Shanghai 200001 China; ^2^ Shanghai Institute of Precision Medicine Shanghai Ninth People's Hospital Shanghai Jiaotong University School of Medicine Shanghai 200120 China

**Keywords:** diabetic cardiomyopathy, lipid toxicity, lipophagy

## Abstract

Diabetic cardiomyopathy (DbCM) is characterized by diastolic dysfunction, which progresses into heart failure and aberrant electrophysiology in diabetic patients. Dyslipidemia in type 2 diabetic patients leads to the accumulation of lipid droplets (LDs) in cardiomyocytes and results in lipid toxicity which has been suggested to drive DbCM. It is aimed to explore potential pathways that may boost LDs degradation in DbCM and restore cardiac function. LDs accumulation resulted in an increase in lipid toxicity in DbCM hearts is confirmed. Microlipophagy pathway, rather than traditional macrolipophagy, is activated in DbCM hearts. RNA‐Seq data and Rab7‐CKO mice implicate that Rab7 is a major modulator of the microlipophagy pathway. Mechanistically, Rab7 is phosphorylated at Tyrosine 183, which allows the recruitment of Rab‐interacting lysosome protein (Rilp) to proceed LDs degradation by lysosome. Treating DbCM mice with Rab7 activator ML‐098 enhanced Rilp level and rescued the observed cardiac dysfunction. Overall, Rab7‐Rilp‐mediated microlipophagy may be a promising target in the treatment of lipid toxicity in DbCM is suggested.

## Graphical abstract

How cardiomyocytes digest excessive lipid droplets and overcome lipid toxicity in diabetic cardiomyopathy remains unknown. Rab7‐Rilp axis‐mediated microlipophagy is activated in diabetic cardiomyocytes to facilitate lipid droplets degradation, ameliorate lipid toxicity, and protect the heart. Treating mice with Rab7 activator ML‐098 enhances Rilp level and rescues cardiac dysfunction in diabetic cardiomyopathy.

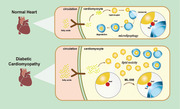

## 1 Introduction

Diabetic cardiomyopathy (DbCM) remains a challenge clinically for diabetes mellitus (DM) patients.^[^ ^1^, ^2^ ^]^ Patients with DbCM exhibit diastolic dysfunction, progression toward systolic dysfunction, abnormal electrophysiology, and are at high risk of developing heart failure.^[^ ^3^ ^]^ The incidence of DbCM has reached 11% in patient of DM and the growing DM patients poses a major challenge to healthcare systems worldwide.^[^ ^4^ ^]^ Currently, it is thought that mitochondrial dysfunction, lipid toxicity, glucotoxicity and oxidative stress play key roles in DbCM progression,^[^ ^5^ ^]^ yet mechanistic insights as to how cardiomyocytes compensate to these stresses remain elusive.

Lipid abnormalities affect 60–70% of type 2 DM (T2DM) patients, resulting in ectopic lipid disposition and cardiovascular diseases.^[^ ^6^, ^7^ ^]^ Using ^1^H‐Magnetic Resonance Spectroscopy, McGavock et al. observed an increase in lipid content in T2DM patient hearts ^[^ ^8^ ^]^ and accumulation of triglyceride lipid droplets (LDs) in cells.^[^ ^9^ ^]^ Although the accumulated LDs can be mobilized to release free fatty acids (FAs) for mitochondria β‐oxidation or membrane synthesis,^[^ ^10^ ^]^ lipid toxicity in mitochondria results in an increase in reactive oxygen species (ROS) and ceramide production.^[^ ^11^, ^12^ ^]^ Whether lipid toxicity drives DbCM remains to be elucidated.

Autophagy is an active intracellular process by which cytoplasmic materials are membrane‐engulfed and fused with lysosomes for degradation.^[^ ^13^ ^]^ Sorting by the process of cargo delivery, autophagy can be classified as macroautophagy (degradation of engulfed cargos via autophagosome fusion), microautophagy (direct fusion of cargo to lysosome), and chaperon‐mediated autophagy (lysosomal degradation of proteins).^[^ ^14^ ^]^ Further, terminology has been extended by the target organelle such as mitophagy (degrading mitochondria), ERphagy (degrading endoplasmic reticulum), and more. Lipophagy, a special form of autophagy that degrades accumulated LDs, has been reported to be prevalent in liver diseases and cancer.^[^ ^15^ ^]^ However, whether lipophagy is present in DbCM remains controversial. In 2019, Tong et al. reported the absence of lipophagy in DbCM based on lack of autophagosome‐engulfed LDs.^[^ ^16^ ^]^ Conversely, Mardani et al. claimed the existence of lipophagy in DbCM by demonstrating colocalization of LDs signals and lysosome marker Lamp1.^[^ ^17^ ^]^ These contradictions need to be better resolved to provide better justification for targeting lipophagy in treating lipotoxicity in DbCM.

Rab7 is a GTPase that participates in membrane‐tethering process with its lysosomal binding partner Rilp (Rab‐interacting lysosome protein), and Rab7‐Rilp is recruited to late endosomes and lysosomes during various forms of autophagy.^[^ ^18^ ^]^ It has been demonstrated that the active form of Rab7 (Rab7 Q67L) is indispensable for LDs breakdown ^[^ ^19^ ^]^ and in its GTP‐bound active form, lysosomal Rab7 can promote mitochondria‐lysosome contact to facilitate mitochondrial degradation.^[^ ^20^ ^]^ The role of Rab7 in DbCM LDs degradation and whether Rab7 participates in DbCM pathogenesis remains unknown.

Using a high fat diet (HFD) coupled to streptozocin (STZ) induced type 2 diabetic mouse model and diabetic human heart sample, we confirm the existence of lipophagy in DbCM. We demonstrate that microautophagic degradation of LDs (microlipophagy), rather than traditional macroautophagic degradation (macrolipophagy), is the dominant LDs degradation pathway in DbCM cardiomyocytes. Molecularly, Tyrosine 183 phosphorylation of Rab7 recruits Rilp and initiates microlipophagy in DbCM cardiomyocytes. Despite constant fatty acids (FAs) stimulation, limited Rilp protein levels limit degree of microlipophagy and results in lipid toxicity in DbCM. Promoting Rilp recruitment by Rab7 activator ML‐098 rescued DbCM cardiac dysfunction. Our findings advance the understanding of microlipophagy and reveal a molecular target to mitigate lipid toxicity in DbCM by targeting the Rab7‐Rilp axis.

## 2 Results

### 2.1 Presence of Lipid Toxicity in DbCM

**Figure 1:**
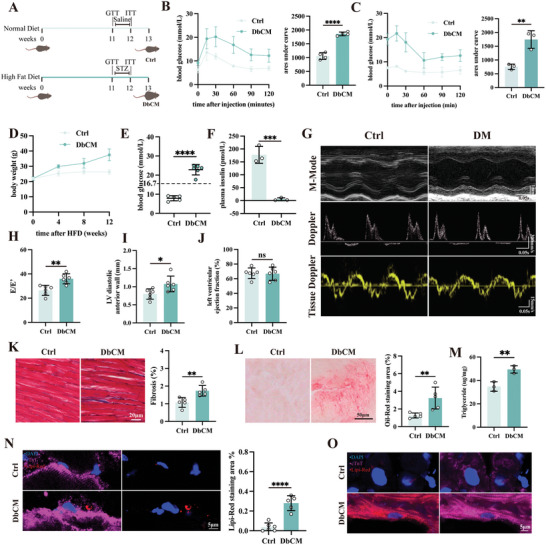
Presence of lipid toxicity in DbCM. A) A flowchart describing the establishment of DbCM mice model. B) Changes in blood glucose level measured after injection of α‐d‐glucose on mice and quantification of areas under curve (*n* = 4 per group). C) Changes in blood glucose level measured after injection of insulin on mice and quantification of areas under curve (*n* = 3 per group). D) Recorded body weight of DbCM mice (*n* = 3–5 per group). E) Random blood glucose of DbCM mice (*n* = 5‐6 per group). F) Plasma insulin level of DbCM mice (*n* = 3 per group). G) Representative micrographs of m‐mode, doppler, and tissue doppler echocardiography. H) Evaluation of diastolic function by E/e’ (*n* = 6 per group). I) Analysis on the thickness of left ventricular diastolic anterior wall (LVAW:d) (*n* = 6 per group). J) Evaluation of systolic function by left ventricular ejection function (*n* = 6 per group). K) Representative micrographs of Masson's trichrome staining in heart sections of mice and quantification on fibrotic areas from Masson's trichrome staining (*n* = 5 per group). L) Representative micrographs of Oil‐Red staining in DbCM cryosection and quantification of Oil‐Red Staining Area (%) (*n* = 5 per group). M) Triglyceride content in DbCM mice hearts (*n* = 3 per group). N) Representative micrographs of immunofluorescence staining of Lipi‐Red to label LDs in mice heart cryosection and quantification of Lipi‐Red staining area (*n* = 5 per group). The cell nuclei were stained with DAPI (blue) and cardiomyocytes were stained with cardiac troponin T (cTnT, magenta). O) Representative micrographs of immunofluorescence staining of Lipi‐Red to label LDs in human heart cryosection. The cell nuclei were stained with DAPI (blue) and cardiomyocytes were stained with cardiac troponin T (cTnT, magenta). The Student's *t*‐test was used to analyze the differences between two groups and the data are expressed as mean ± SD, ns: no significance, ^*^ *p* < 0.05, ^**^ *p* < 0.01, ^***^ *p* < 0.001 and ^****^ *p* < 0.0001).

To study lipid toxicity in diabetic murine hearts, an established DbCM model where C57BL/6J mice were fed with a high‐fat diet (HFD) for three months followed by STZ challenge to induce insulin resistance was used (**Figure** **1**A). Using the glucose tolerance test (GTT) and insulin tolerance test (ITT), we confirm that our HFD mice were intolerant to glucose challenge and were deficient in handling elevated glucose compared to the control group (Figure 1B,C). Changes in body weight, unstable blood glucose levels, and insulin levels were observed in DbCM animals (Figure 1D–F). Compared to control animals, DbCM animals exhibited diastolic dysfunction (reflected by an increase in E/e’ but not E/A waves ratio, Figure 1G,H; Figure S1A, Supporting Information), increased anterior wall thickness (LVAW:d) (Figure 1I), and attenuated global longitudinal strain (GLS) (Figure S1B, Supporting Information), but no significant changes in left ventricular ejection fraction (LVEF) and posterior wall thickness (LVPW:d, Figure 1J; Figure S1C, Supporting Information). To evaluate whether the observed diastolic dysfunction is accompanied with extensive fibrosis, we subjected DbCM heart sections with Masson staining and confirmed the presence of severe cardiac fibrosis (Figure 1K). Besides, DbCM hearts show an overexpression of genes associated with hypertrophy (Myh7, Nppa, Nppb), fibrosis (Col1a1, Col3a1), and inflammation (Tnf, Il‐6) (Figure S1D–F, Supporting Information). Next, we Langendorff purified adult murine ventricular myocytes (AMVMs) and measured contractile function using the Ionoptix HTS system. At single cardiomyocyte level, a significant decrease in fractional shortening and diastolic velocity was observed in DbCM AMVMs compared to control AMVMs (Figure S1G–J, Supporting Information), confirming the presence of diastolic dysfunction. Systolic function, however, remains unchanged (Figure S1K,L, Supporting Information).

To study whether this cardiac dysfunction is induced by lipid toxicity in DbCM, we first examine whether FAs uptake is altered in our DbCM murine hearts. As shown in Figure S2A (Supporting Information), genes responsible for FAs uptake, Cd36, and Fabp3, were significantly elevated in DbCM AMVMs compared to controls. Next, we examined the lipolysis pathway by measuring the expression levels of Atgl (adipose triglyceride lipase) and Hsl (hormone sensitive lipase) in DbCM. As shown in Figure S2B (Supporting Information), level of Atgl and Hsl did not significantly changed in DbCM mice compared to the control, indicating unaltered DbCM lipolysis. Last, we examined mitochondrial oxidation capacity. Despite attenuated peak oxygen consumption rate (OCR), basal OCR was not significantly altered (Figure S2C–E, Supporting Information). Together, these results demonstrate increased FAs uptake, but no change in breakdown and utilization, in DbCM cardiomyocytes.

Next, to verify LDs accumulation, we stained DbCM cryosection with Oil‐Red Staining and Lipi‐Red probe (staining LDs), and measured triglyceride (TG) content in DbCM hearts where we confirmed LDs accumulation (Figure 1L–N). Moreover, we also confirmed LDs accumulation in type 2 DM patient's heart (Figure 1O). To confirm whether this LDs accumulation presented toxicity in DbCM heart, we examine signatures of lipid toxicity including intracellular ROS level and ER stress.^[^ ^11^ ^]^ As shown in Figure S2F,G (Supporting Information), Langendroff isolated AMVMs from DbCM mice exhibited an increase in ROS level and ER stress marker expression (Atf6 and Ire1α), suggesting significant lipid toxicity. All these results confirmed LDs accumulation and lipid toxicity in DbCM.

To understand the cause of lipid toxicity, we constructed an in vitro DbCM model by treating isolated neonatal mouse cardiomyocytes (NMCMs) with high glucose supplemented with BSA‐conjugated palmitate acid media (HGPA, Figure S3A, Supporting Information),^[^ ^21^, ^22^ ^]^ where palmitate‐free BSA media was used as control (Ctrl). In FAs‐treated NMCMs, we observed an increase in LDs accumulation (Figure S3B, Supporting Information), TG content (Figure S3C, Supporting Information), and molecular evidence of hypertrophy and inflammation (Figure S3D,E, Supporting Information). By tracing NMCMs contraction (Figure S3F, Supporting Information), we found that the contractile function of NMCMs deteriorated in a time‐dependent manner with HGPA culture conditions (Figure S3G, Supporting Information). Since no toxicity was found in Ctrl media compared to DMEM (Figure S3H, Supporting Information), the above results implicate that LDs accumulation correlates with contractile dysfunction and cardiac toxicity. Further, increased cellular ROS levels and ER stress were present in HGPA‐treated NMCMs (Figure S3I,J, Supporting Information). Together, we confirmed DbCM lipid toxicity both in vivo and in vitro.

### 2.2 Induction of Macroautophagy Does Not Prevent LDs Accumulation in DbCM

**Figure 2:**
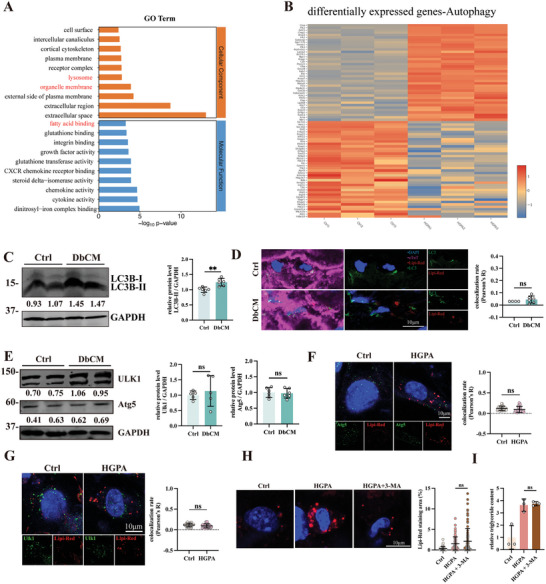
Induction of macroautophagy does not prevent LDs accumulation in DbCM. A) GO analysis on enrichments of differentially expressed genes (DEGs). B) Top upregulated and downregulated DEGs associated with autophagy. C) Representative blots, relative intensity and quantitative analysis of immunoblots analysis of LC3B in Langendroff AMVMs (*n* = 6 per group). D) Representative micrographs of immunofluorescence double‐staining of LC3 to label autophagosome and Lipi‐Red (Red) to label LDs, and quantification of Lipi‐Red colocalizing LysoTracker, results were presented as Pearson’ R (*n* = 4‐6 per group). The cell nuclei were stained with DAPI (blue) and cardiomyocytes were stained with cardiac troponin T (cTnT, magenta). E) Representative blots, relative intensity, and quantitative analysis of immunoblots analysis of Ulk1 and Atg5 in Langendroff AMVMs (*n* = 5‐6 per group). F) Representative micrographs of immunofluorescence double‐staining of Lipi‐Red (red) to label LDs and Atg5 (green), and quantification of Lipi‐Red colocalizing Atg5, results were presented as Pearson’ R (at least *n* = 30 from 3 individual tests per group). The cell nuclei were stained with DAPI (blue). G) Representative micrographs of immunofluorescence double‐staining of Lipi‐Red (red) to label LDs and Ulk1 (green), and quantification of Lipi‐Red colocalizing Ulk1, results were presented as Pearson’ R (at least *n* = 30 from 3 individual tests per group). The cell nuclei were stained with DAPI (blue). H) Representative micrographs of immunofluorescence staining of Lipi‐Red (red) to label LDs and quantification of Lipi‐Red staining area (at least *n* = 40 from three individual tests per group). The cell nuclei were stained with DAPI (blue). I) Quantification of relative triglyceride content in NMCMs after 3‐MA treatment (*n* = 3 per group). The Student's *t*‐test was used to analyze the differences between two groups and the data are expressed as mean ± SD, ns: no significance and ^**^ *p* < 0.01).

Given FAs import is increased while utilization remains unchanged in DbCM cardiomyocytes, we asked if enhancing LDs degradation can prevent the onset of contractile dysfunction. To determine which is the main lipid degradation pathway in DbCM, we performed RNA‐Seq analysis on HGPA‐treated and Control HL‐1 cells. Analysis showed that a great proportion of differentially expressed genes (DEGs) enriched in pathways of lysosome and organelle membrane (**Figure** **2**A). Lysosome is the key organelle for autophagic degradation. Differential gene analysis showed a large proportion of autophagic genes are differentially expressed between our HGPA and control HL‐1 (Figure 2B). Taken together, our RNA‐Seq analysis suggests that dysregulated autophagy may be responsible for LDs accumulation in DbCM.

To evaluate degree of autophagic capacity, we first examined LC3B protein levels in Langendorff purified cardiomyocytes. Compared to control cardiomyocytes, DbCM cardiomyocytes exhibited an increase in LC3B protein levels (Figure 2C). Next, to see whether this increase in LC3B‐II level is suggestive of macroautophagy, we performed immunofluorescence colocalization staining. To our surprise, we observed no difference in LC3B‐LDs colocalization between DbCM and control cardiomyocytes (Figure 2D). To test whether other macroautophagy regulators are associated with DbCM lipophagy, we measured two macroautophagy markers, Ulk1 and Atg5. Levels of Ulk1 and Atg5 showed no difference between DbCM and control hearts (Figure 2E), and localization to LDs surfaces in HGPA‐treated NMCMs were absent (Figure 2F,G). Further, inhibition of macroautophagy by the addition of 3‐Methyladenine (3‐MA, 5 mm) did not alter LDs accumulation (Figure 2H,I).

Autophagy is a dynamic degradation process. To explore whether LDs accumulation in DbCM is caused by a blocked autophagy flux, we first examined level of p62, an autophagy degradation indicator, in DbCM AMVMs. As shown in Figure S4A (Supporting Information), we found no significant changes in the p62 level, implicating autophagy flux may not be blocked in DbCM. To verify our conclusion, we transfected RFP‐GFP‐LC3 lentivirus to HL‐1 cardiomyocytes to dynamically observe autophagy flux. In this system, autophagosome emit both red and green (yellow) flurorences, while autolysosome (autophagosome fusing with lysosome) emit red flurorences only. According to our results, both autophagosome and autolysosome signals increase when treating HGPA, which supports that autophagy flux is not blocked in our in vitro DbCM model (Figure S4B, Supporting Information). Together, these results suggest that macroautophagy, as well as blocked autophagy flux, does not take part in myocardial LDs accumulation and degradation.

### 2.3 Microlipophagy Dominates LDs Degradation in DbCM

**Figure 3:**
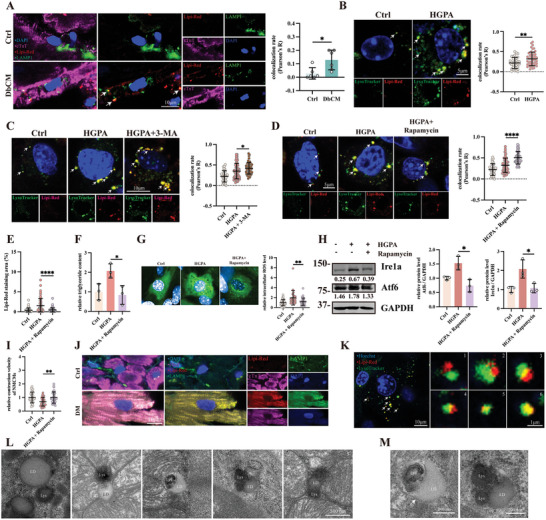
Microlipophagy dominates LDs degradation in DbCM. A) Representative micrographs of immunofluorescence double‐staining of Lipi‐Red (red) to label LDs and Lamp1 (green) to label lysosome, and quantification of Lipi‐Red colocalizing Lamp1, results were presented as Pearson’ R (*n* = 6 per group). The cell nuclei were stained with DAPI (blue) and cardiomyocytes were stained with cardiac troponin T (cTnT, magenta). B) Live‐cell confocal imaging of Ctrl and HGPA treated NMCMs incubated Lipi‐Red (red) to label LDs and Lysotracker (green) to label lysosome, and quantification of Lipi‐Red colocalizing LysoTracker, results were presented as Pearson’ R (at least 30 cells per group from three individual tests). Cell nuclei were labeled with Hoechst 33342 and arrows indicate examples of Lipi‐Red overlapping LysoTracker. C) Confocal micrographs using the same assay conditions as in (B) after 3‐MA treatment, and quantification of Lipi‐Red colocalizing LysoTracker, results were presented as Pearson’ R. (at least 30 cells per group from three individual tests). Cell nuclei were labeled with Hoechst 33 342 and arrows indicate examples of Lipi‐Red overlapping LysoTracker. D) Confocal micrographs after Rapamycin treatment using the same assay conditions as in (B) and quantification of Lipi‐Red colocalizing LysoTracker, results were presented as Pearson’ R (at least 30 cells per group from three individual tests). Cell nuclei were labeled with Hoechst 33342 and arrows indicate examples of Lipi‐Red overlapping LysoTracker. E) Quantification of Lipi‐Red Staining Area (%) in Ctrl, HGPA, and HGPA+Rapamycin treated NMCMs (at least 40 cells from three individual tests per group f). F) Quantification of relative triglyceride content in NMCMs after Rapamycin treatment (*n* = 3 per group). G) Representative micrograph and quantitative analysis of intracellular reactive oxygen species (ROS) level in NMCMs after Rapamycin treatment (at least n = 20 from 3 individual tests per group). H) Representative blots, relative intensity, and quantitative analysis of immunoblots analysis of Atf6 and Ire1α in Rapamycin treated NMCMs (*n* = 6 per group). I) Relative contraction velocity of NMCMs after Rapamycin treatment (at least 30 cells per group from 3 individual tests). J) Representative micrographs of immunofluorescence double‐staining of Lipi‐Red (red) to label LDs and Lamp1 (green) to label lysosome in human heart section. The cell nuclei were stained with DAPI (blue) and cardiomyocytes were stained with cardiac troponin T (cTnT, magenta). K) Confocal micrographs using the same assay conditions as in (B), presenting a gradual fusion of LDs to lysosome from 1–6. L) Transmission electron microscopy (TEM) micrographs from DbCM mice heart. LDs refers to lipid droplets. Lys refers to lysosome. Sequence 1–5 indicate a fusion process between LDs and lysosome. M) Zoomed TEM micrographs from J. Arrows indicates that there are no double‐membrane structures exist. The Student's *t*‐test was used to analyze the differences between two groups and the data are expressed as mean ± SD, ^*^ *p* < 0.05, ^**^ *p* < 0.01, and ^****^ *p* < 0.0001).

Since we failed to observe autophagosome recruitment to LDs surface in DbCM, we sought to determine whether LDs can be targeted to lysosome directly in DbCM hearts. We first looked for co‐localization of lysosome (Lamp1) with LDs (Lipi‐Red) in DbCM hearts. As shown in **Figure** **3**A, DbCM murine hearts exhibit a significant increase in colocalization of LDs and Lamp1 compared to control hearts, suggesting active LDs transportation to lysosomes. Next, we stained HGPA‐treated NMCMs with LysoTracker (live cell dye for lysosome) and Lipi‐Red (for LDs) to assay LDs‐lysosome recruitment in vitro. In accordance to DbCM heart section results, LDs recruitment to lysosome is significantly upregulated in HGPA‐treated NMCMs compared to controls (Figure 3B). To determine whether this recruitment occurs through the formation of autophagosome, we treated HGPA‐NMCMs with 3‐MA (5 mm) to inhibit autophagosome formation. Consistently, inhibiting autophagosome formation didn't attenuate LDs recruitment to lysosome (Figure 3C). Together, these data demonstrate that LDs can be sent to lysosome without the recruitment of autophagosome.

To confirm whether colocalization of LDs with lysosomes leads to lipophagy, we induced lysosomal activity by Rapamycin (50 µm) in our HGPA treated NMCMs. Indeed, Rapamycin treatment increased colocalization of LDs with lysosome (Figure 3D), decreased LDs accumulation (Figure 3E,F), decreased ROS level (Figure 3G), alleviated ER stress (Figure 3H) and improved NMCMs contractility (Figure 3I). To confirm the presence of lipophagy in diabetic patient hearts, we stained diabetic patient hearts with markers of lysosome (Lamp1) and LDs and confirmed the presence of active lipophagy in DbCM (Figure 3J). Taken together, our results suggest the presence of lipophagy in murine and patient DbCM hearts.

To further elucidate how LDs are trafficked to lysosome independent of autophagosomes, we examined whether microautophagy, a direct fusion between cargos and lysosome, is activated in DbCM cardiomyocytes. To determine whether microlipophagy exists in DbCM, we stained HGPA‐treated NMCMs with markers of LDs (Lipi‐Red) and lysosome (LysoTracker) to observe whether these two compartments directly contact. As shown in Figure 3K, we observed colocalization between LDs and lysosome (sequence 1–6 in Figure 3K). To further confirm our hypothesis, we used transmission electron microscopy (TEM) on DbCM and control murine hearts. Compared to control hearts, we observed LDs‐lysosome contact and engulfment suggestive of microlipophagy (sequence 1–5 in Figure 3L). Moreover, the engulfment of LDs is free of double‐membrane autophagosome (Figure 3M). Taken together, our results suggested that autophagosome independent microlipophagy is the dominant form of lipophagy in DbCM.

### 2.4 Rab7‐Rilp Axis Mediate Microlipophagy in DbCM

**Figure 4:**
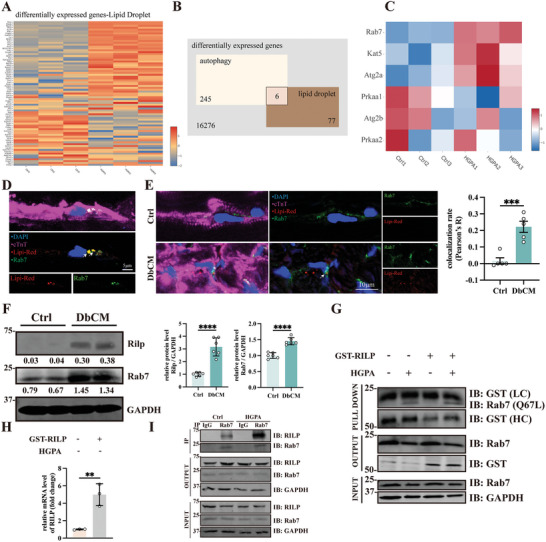
Rab7‐Rilp axis mediate microlipophagy in DbCM. A) All DEGs that were associated with LDs within the group Ctrl and HGPA from RNA‐Seq analysis. B) Flowchart representing the analysis of DEGs. Among all 16 276 DEGs, there are 245 genes associated with autophagy and 77 associated with LDs, while only 6 DEGs are associated with both. C) Expressions of statistically significant DEGs that were associated both with autophagy and LDs. D) Confocal micrographs of human heart samples stained with Rab7 (Green) and Lipi‐Red (Red). The cell nuclei were stained with DAPI (blue) and cardiomyocytes were stained with cardiac troponin T (cTnT, magenta). E) Representative micrographs of immunofluorescence staining of Rab7 (green) and Lipi‐Red (red) to label LDs in DbCM mice heart and quantification of Lipi‐Red colocalizing Rab7. Results were presented as Pearson’ R (*n* = 5 per group). The cell nuclei were stained with DAPI (blue) and cardiomyocytes were stained with cardiac troponin T (cTnT, magenta). F) Representative blots, relative intensity, and quantitative analysis of immunoblots analysis of Rab7 and Rilp in DbCM AMVMs (*n* = 6 per group). G) Representative blots of GST interacted Rab7 (Q67L) in HL‐1 cells transfected GST‐Rilp. H) Relative mRNA expression of GST‐Rilp in HL‐1 cells transfected with GST‐Rilp. I) Representative blots of Rab7 immunoprecipitated Rilp in Ctrl and HGPA group. The Student's *t*‐test was used to analyze the differences between two groups and the data are expressed as mean ± SD, ^**^ *p* < 0.01, ^***^ *p* < 0.001 and ^****^ *p* < 0.0001).

To interrogate the mechanistic underpinnings of microlipophagy, we analyzed DEGs in our RNA‐Seq analysis that is associated with both LDs (**Figure** **4**A) and autophagy. Among 16 276 DEGs, there were 245 genes associated with autophagy and 77 genes associated with LDs, while only 6 genes associated with both (Figure 4B). We summarized all 6 DEGs and find that Rab7 is the only gene with statistical significance under HGPA treatment (Figure 4C; Table S1, Supporting Information). Rab7 is a GTPase that is previously believed to mediate organelle fusion by acting with its downstream effector Rab‐interacting lysosome protein (Rilp).^[^ ^23^ ^]^ While the role of Rab7 in DbCM remains unknown. First, we evaluated the colocalization of Rab7 and LDs in DM patient hearts and DbCM mice hearts. By immunofluorescence staining we observed a strong colocalization signal between Rab7 and LDs in both patient and murine DbCM cardiomyocytes (Figure 4D,E). We then sought to determine whether the observed microlipophagy in DbCM is mediated by Rab7 and this downstream Rilp.

To study the role of Rab7‐Rilp axis in DbCM, we first measured the expression changes of Rab7 and Rilp in Langendroff purified DbCM cardiomyocytes. Indeed, DbCM cardiomyocytes exhibit a significant increase in Rab7 and Rilp protein expression (Figure 4F). It has been demonstrated that activated Rab7 (or Rab7 Q67L) can bind to Rilp to initiate cargo transportation to lysosome.^[^ ^23^ ^]^ To study this interaction, in GST‐tagged Rilp overexpressing HL‐1 cardiomyocytes, HGPA treatment led to increased active‐Rab7 association when we performed anti‐GST immunoprecipitation (Figure 4G,H). The reverse immunoprecipitation using anti‐Rab7 antibody also showed enhanced association with Rilp protein in HGPA‐treated HL‐1 cardiomyocytes (Figure 4I). Taken together, the above results support that DbCM condition induces cardioprotective microlipophagy through Rab7‐Rilp pathway.

### 2.5 Microlipophagy can be Manipulated by Targeting Rab7‐Rilp axis

**Figure 5:**
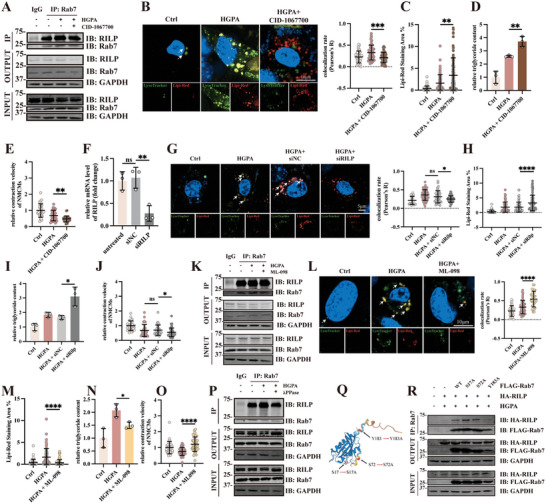
Microlipophagy can be manipulated by targeting Rab7‐Rilp axis. A) Representative blot of Rab7 immunoprecipitated Rilp after treatment of CID‐1067700. B) Live‐cell confocal imaging of Ctrl, HGPA, and HGPA + CID‐1067700 treated NMCMs incubated Lipi‐Red (red) to label LDs and Lysotracker (green) to label lysosome, and quantification of Lipi‐Red colocalizing LysoTracker, results were presented as Pearson’ R (at least 30 cells from three individual tests per group). Cell nuclei were labeled with Hoechst 33342 and arrows indicate examples of Lipi‐Red overlapping LysoTracker. C) Quantification of Lipi‐Red Staining Area (%) in Ctrl, HGPA, and HGPA+CID‐1067700 treated NMCMs (at least 30 cells per group from three individual tests). D) Quantification of relative triglyceride content in NMCMs after CID‐1067700 treatment (n = 3 per group). E) Relative contraction velocity of Ctrl, HGPA, and HGPA+CID‐1067700 incubated NMCMs (at least 30 cells per group from three individual tests). F) Relative mRNA expression of Rilp in HL‐1 cells transfected with siRilp. G Representative micrographs of immunofluorescence staining of Lysotracker (Green) and Lipi‐Red (Red) in cells transfected with siRilp, and quantification of Lipi‐Red colocalizing LysoTracker, results were presented as Pearson’ R (at least 30 cells per group from 3 individual tests). H) Quantification of Lipi‐Red Staining Area (%) in Ctrl, HGPA, HGPA with or without siRilp transfected NMCMs (at least 30 cells per group from 3 individual tests). I) Quantification of relative triglyceride content in NMCMs after siRilp transfection (*n* = 3 per group). J) Relative contraction velocity of Ctrl, HGPA, HGPA with or without siRilp transfected treated in NMCMs (at least 30 cells per group from 3 individual tests). K) Representative blot of Rab7 immunoprecipitated Rilp after treatment of ML‐098. L) Live‐cell confocal imaging of Ctrl, HGPA, and HGPA + ML‐098 treated NMCMs incubated Lipi‐Red (red) to label LDs and Lysotracker (green) to label lysosome and quantification of Lipi‐Red colocalizing LysoTracker, results were presented as Pearson’ R. Cell nuclei were labeled with Hoechst 33 342, arrows indicate examples of Lipi‐Red overlapping LysoTracker. M Quantification of Lipi‐Red Staining Area (%) in Ctrl, HGPA, and HGPA+ML‐098 treated NMCMs (at least 30 cells per group from 3 individual tests). N) Quantification of relative triglyceride content in NMCMs after ML‐098 treatment (*n* = 3 per group). O) Relative contraction velocity of NMCMs after ML‐098 treatment (at least 30 cells per group from 3 individual tests). P) Representative blot of Rab7 immunoprecipitated Rilp after treatment of lambda‐PPase. Q) Structure of Rab7 with Serine 17, Serine 72, and Tyrosine 183 highlighted. R) Representative blot of FLAG‐Rab7 immunoprecipitated HA‐Rilp after mutations of the targeted amino acid. The Student's t‐test was used to analyze the differences between two groups and the data are expressed as mean ± SD, ns: no significance, ^*^ *p* < 0.05, ^**^ *p* < 0.01, ^***^ *p* < 0.001 and ^****^ *p* < 0.0001).

Given that Rab7‐Rilp mediated microlipophagy is induced in DbCM, we asked whether targeting Rab7‐Rilp axis affects microlipophagy. Given Rab7 is a GTPase, we treated HGPA‐treated NMCMs with a Rab7‐specific inhibitor CID‐1067700 (40 µm). Treatment of CID‐1067700 significantly impaired Rab7‐Rilp interaction, as shown by less Rab7 interacted Rilp (**Figure** **5**A). Further, CID‐1067700 treatment significantly impaired LDs recruitment to lysosome, suggesting an attenuated lipophagy level (Figure 5B) and results in accumulation of LDs (Figure 5C‐D), leading to increased cellular ROS level (Figure S5A, Supporting Information), ER stress (Figure S5B, Supporting Information) in cells, and contractile dysfunction (Figure 5E). In support, Rilp knockdown (Figure 5F) also resulted in an increase in LDs accumulation (Figure 5G–I), ROS level (Figure S5C, Supporting Information), ER stress (Figure S5D, Supporting Information), and impaired cardiac contractility (Figure 5J). Together, those results indicate that disruption of Rab7‐Rilp activation blocks microlipophagy and results in LDs accumulation and impaire contractility.

Next, we explored whether activating Rab7 GTPase activity, by a Rab7 specific activator ML‐098 (0.5 µm), helps degrade LDs and counteract lipid toxicity in DbCM. ML‐098 treatment resulted in an increase in Rilp recruitment by Rab7 (Figure 5K), upregulation of LDs‐lysosome colocalization (Figure 5L), decrease in LDs accumulation (Figure 5M,N), ROS level (Figure S5E, Supporting Information), ER stress (Figure S5F, Supporting Information) and improved contractile function (Figure 5O). Further, we confirm that siRilp treatment was sufficient in ablating the effects of ML‐098 (Figure S5G, Supporting Information).

Rab7 has been previously discovered that can be activated by phosphorylation.^[^ ^24^ ^]^ To examine whether Rab7 phosphorylation is necessary for microlipophagy activation, we subjected HGPA NMCMs with a phosphorylation inhibitor, λ‐PPase (lambda‐phosphatase). Inhibition of cellular phosphorylation significantly reduces Rilp recruitment by Rab7 (Figure 5P) under HGPA stimulation. Given that Rab7 can be phosphorylated at Serine 17, Serine 72 or Tyrosine 183,^[^ ^24^, ^25^, ^26^ ^]^ we overexpressed these phosphor‐null mutants in HEK 293T cells and evaluated their ability to recruit Rilp (Figure 5Q). Among these three mutants, only Y183A mutant exhibited a loss of its ability to interact with Rilp (Figure 5R). To confirm the role of Rab7 tyrosine phosphorylation in DbCM, we immnunopercipitated Rab7 and immunoblotted with an anti‐phosphotyrosine antibody. Rab7 tyrosine phosphorylation level significantly increased under HGPA treatment, which can be abolished by Rab7 inhibitor CID‐1067700 or enhanced by treatment of ML‐098, respectively (Figure S6A–C, Supporting Information). Taken together, our results demonstrate that Rab7, via Y183 tyrosine phosphorylation, recruits Rilp and activates microlipophagy in DbCM.

To confirm the essential role of Rab7 Y183 phosphorylation in lipophagy, we constructed a Rab7^Y183A^ lentivirus and transfected NMCMs to study lipid recycling in vitro. Rab7^Y183A^ overexpression significantly abolished lipophagy in HGPA‐treated NMCMs (Figure S6D, Supporting Information). This resulted in a significant increase in LDs accumulation (Figure S6E,F, Supporting Information), ROS levels (Figure S6G, Supporting Information), ER stress (Figure S6H, Supporting Information) and impaired contractility (Figure S6I, Supporting Information). These observations suggest blocked lipophagy response results in an exacerbated lipid accumulation and lipid toxicity in HGPA treated cardiomyocytes.

### 2.6 Rab7‐CKO Mice Present Attenuated Lipophagy and More Severe Lipid Toxicity

**Figure 6:**
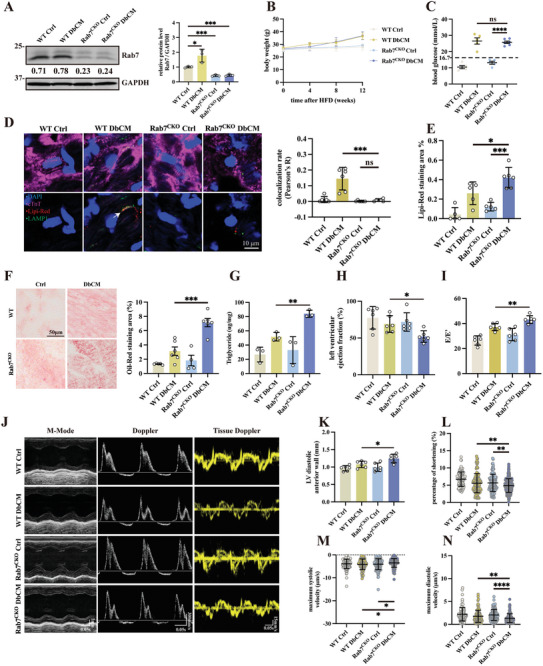
Rab7‐CKO mice present attenuated lipophagy and severe lipid toxicity. A) Representative image, relative intensity, and quantification of immunoblots analyzing Rab7 in WT and Rab7‐CKO mice respectively establishing DbCM model (*n* = 3 per group). B) Recorded body weight changes of DbCM mice (*n* = 5 per group). C) Random blood glucose of DbCM mice (*n* = 5‐6 per group). D) Representative micrographs of immunofluorescence double‐staining of Lipi‐Red (red) to label LDs and Lamp1 (green) to label lysosome, and quantification of Lipi‐Red colocalizing Lamp1, results were presented as Pearson’ R (*n* = 6 per group). The cell nuclei were stained with DAPI (blue) and cardiomyocytes were stained with cTnT (magenta). Arrows indicate examples of Lipi‐Red overlapping Lamp1. E) Quantification of Lipi‐Red Staining Area (%) (*n* = 5‐6 per group) in WT and Rab7‐CKO mice. F) Representative micrographs of Oil‐Red staining in DbCM cryosection and quantification of Oil‐Red Staining Area (%) (*n* = 4‐6 per group). G) Quantification of triglyceride content in DbCM hearts (*n* = 3 per group). H) Evaluation of systolic function by left ventricular ejection function (*n* = 6 per group). I) Evaluation of diastolic function by E/e’ (*n* = 6 per group). J) Representative micrographs of m‐mode, doppler, and tissue doppler echocardiography. K) Evaluation of left ventricular diastolic anterior wall thickness (LVAW:d) (*n* = 6 per group). Evaluation on L) percentage of shortening, M) maximum systolic velocity, and N) maximum diastolic velocity in Langendorff‐isolated adult mouse cardiomyocytes (AMCMs) (at least 90 cells from three individual mice per group). The Student's *t*‐test was used to analyze the differences between two groups and the data are expressed as mean ± SD, ns: no significance, ^*^ *p* < 0.05, ^**^ *p* < 0.01, ^***^ *p* < 0.001 and ^****^ *p* < 0.0001).

To study how Rab7 and Rab7 mediated microlipophagy impact cardiac function in vivo, we generated Rab7 cardiac‐specific knockout (Rab7^CKO^) mice using Rab7^flox/flox^ crossed with tamoxifen‐inducible Myh6‐Cre^ERT2^ recombinase system (**Figure** **6**A; Figure S7A, Supporting Information) induced DbCM (Figure S7B, Supporting Information). Compared to controls, Rab7^CKO^ DbCM animals did not exhibit significant changes in body weights, random blood glucose, GTT, ITT and plasma insulin level (Figure 6B‐C; Figure S7C–F, Supporting Information). Rab7 deficiency worsened lipophagy in DbCM as evidenced by loss of lysosome (Lamp1) and LDs (Lipi‐Red) colocalization in DbCM hearts (Figure 6D), and resulted in exacerbated LDs accumulation (Figure 6E‐G). Upon diet challenge, Rab7^CKO^ DbCM mice exhibited systolic and diastolic dysfunction (Figure 6H–J; Figure S8A,B, Supporting Information), increased LVAW:d (Figure 6K), and exacerbated GLS (Figure S8C, Supporting Information). However, Rab7^CKO^ mice with normal diet (Rab7^CKO^ Ctrl) showed normal systolic and diastolic functions (Figure 6H–K; Figure S8A–C, Supporting Information) suggesting loss of Rab7 does not affect cardiac function at homeostasis. Histologically, Rab7^CKO^ DbCM mice have a more severe cardiac fibrosis comparing to WT DbCM mice (Figure S8D, Supporting Information). Molecular evidence of cardiomyopathy, including inflammation marker (Tnd and Il6), fibrosis marker (Col1a1 and Col3a1), and hypertrophy marker (Nppa, Nppb, and Myh7), significantly increases when Rab7 was knocked out (Figure S8E–G, Supporting Information). At cellular level, Langendorff isolated AMVMs from Rab7^CKO^ DbCM mice showed the impaired percentage of shortening, diastolic function (by evaluating maximum diastolic velocity and time to maximum diastolic velocity), and systolic function (by evaluating maximum systolic velocity and time to maximum systolic velocity) comparing to Rab7^CKO^ Ctrl (Figure 6L–N; Figure S8H–J, Supporting Information). Finally, as implications of lipid toxicity, cellular ROS level (Figure S8K, Supporting Information) and ER stress (Figure S8L, Supporting Information) increased in Rab7 ^CKO^ DbCM mice.

We have previously shown that macrolipophagy is not involved in DbCM LDs degradation, while whether it is activated in Rab7‐deficient hearts should be determined. To account whether macroautophagy can be compensatorily activated in Rab7^CKO^ DbCM mice, we first measured the expression changes of macroautophagy marker LC3B, Ulk1, and Atg5 in Rab7^CKO^ mice. We discovered that LC3B, instead of Ulk1 and Atg5, is upregulated in Rab7^CKO^ DbCM mice compared to WT DbCM mice (Figure S9A, Supporting Information). We stained for colocalization of LC3B and LDs signals and found no recruitment suggesting lack of macroautophagy compensation in Rab7^CKO^ DbCM hearts (Figure S9B, Supporting Information). Together, all of these results suggest that Rab7‐deficient mice accumulate worsened LDs upon diet challenge due to impaired lipophagy, and finally led to severe cardiac dysfunction in diabetes.

### 2.7 Activating Rab7 by ML‐098 improves Cardiac Function in DbCM Mice

**Figure 7:**
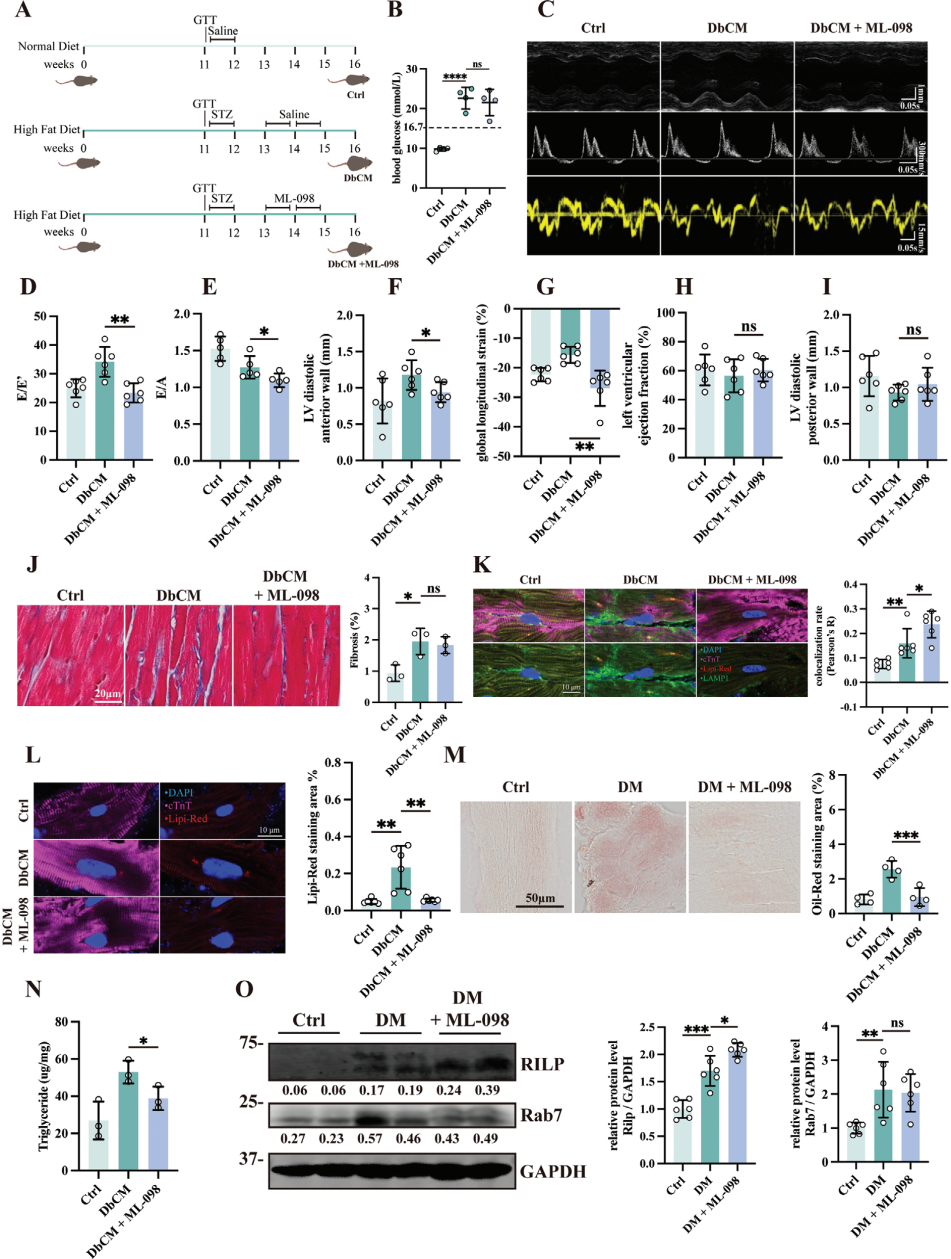
Activating Rab7 by ML‐098 improves cardiac function in DbCM mice. A) A flowchart describing the establishment of DbCM mice model and injection of ML‐098. B) Random blood glucose of DbCM mice (*n* = 5‐6 per group). C) Representative micrographs of m‐mode, doppler, and tissue doppler echocardiography. D) Evaluation of diastolic function by E/e’ (*n* = 6 per group). E) Evaluation of E/A waves ratio. (*n* = 5 per group). F) Analysis on thickness of left ventricular diastolic anterior wall (LVAW:d) (*n* = 6 per group). G) Evaluation on global longitudinal strain (GLS) (*n* = 6 per group). H) Evaluation of systolic function by left ventricular ejection function (*n* = 6 per group). I) Analysis on thickness of left ventricular diastolic posterior wall (LVPW:d) (*n* = 6 per group). J) Representative micrographs of Masson's trichrome staining in heart sections of mice and quantification on fibrotic areas (*n* = 3 per group). K) Representative micrographs of immunofluorescence double‐staining of Lipi‐Red (red) to label LDs and Lamp1 (green) to label lysosome and quantification of Lipi‐Red colocalizing Lamp1, and quantification of Lipi‐Red colocalizing Lamp1, results were presented as Pearson’ R (*n* = 6 per group). The cell nuclei were stained with DAPI (blue) and cardiomyocytes were stained with cardiac troponin T (cTnT, magenta). I) Representative micrographs of immunofluorescence staining of Lipi‐Red to label LDs in mice heart cryosection, and quantification of Lipi‐Red Staining Area (%) in (I) (*n* = 6 per group). The cell nuclei were stained with DAPI (blue) and cardiomyocytes were stained with cardiac troponin T (cTnT, magenta). M) Representative micrographs of Oil‐Red staining in DbCM cryosection and quantification of Oil‐Red Staining Area (%) (*n* = 8 per group). N) Quantification of triglyceride content in DbCM hearts (*n* = 3 per group). O) Representative blots, relative intensity, and quantitative analysis of immunoblots analysis of Rilp and Rab7 in Langendroff AMVMs from groups Ctrl, DbCM, and DbCM treated ML‐098. (*n* = 6 per group). The Student's *t*‐test was used to analyze the differences between two groups and the data are expressed as mean ± SD, ns: no significance, ^*^ *p* < 0.05, ^**^ *p* < 0.01, ^***^ *p* < 0.001, and ^****^ *p* < 0.0001).

We've observed that Rab7‐Rilp mediated lipophagy is the main pathway in DbCM and that LDs accumulation in DbCM contributes to cardiac dysfunction. Altough Rab7 induced lipophagy is insufficient in preventing LDs accumulation, we did not observe a negative feedback loop where HGPA treatment induced Rab7 protein degradation (Figure S10A, Supporting Information). With 0–48 h of HGPA stimulation on HL‐1 cardiomyocytes, we confirmed increased Rab7‐Rilp interaction peaks at 24 h (Figure S10B, Supporting Information). To test whether enhanced Rilp recruitment in DbCM mice can rescue LDs accumulation and lipid toxicity, we *i.p*. injected ML‐098 to stimulate Rilp recruitment in our DbCM mice (**Figure** **7**A). ML‐098 treatment (five consecutive days a week for two weeks) had no effect on body weight, random blood glucose levels, GTT, ITT nor plasma insulin level (Figure 7B; Figure S11A–D, Supporting Information). Interestingly, lipophagy activation by ML‐098 significantly improved diastolic function (E/E’ ratio and E/A ratio), LV diastolic anterior wall thickness, and global longitudinal strain (Figure 7C–G); LV ejection fraction and LV diastolic posterior wall thickness remain unchanged (Figure 7H,I). Histologically, ML‐098 treated hearts displayed no significant changes in cardiac fibrosis (Figure 7J) although it significantly blocked the transcription of fibrotic, inflammatory, and hypertrophic genes (Figure S11E–G, Supporting Information). At cellular level, ML‐098 rescued myocardial percentage of shortening and diastolic function (as represented by maximum diastolic velocity and time to maximum diastolic velocity) (Figure S11H–J, Supporting Information) but had no effect on baseline sarcomeric length and systolic function (Figure S11K–M, Supporting Information).

To confirm ML‐098 efficacy is through myocardial lipophagy activation and lipid toxicity alleviation, we stained DbCM hearts for LDs and lysosome co‐localization. As shown in Figure 7K, colocalization of Lipi‐Red (LDs) and Lamp1 (lyososome) was increased in ML‐098 group and this enhancement correlated with a reduction in LDs accumulation (Figure 7L–N), cellular ROS level (Figure S11N, Supporting Information) and ER stress (Figure S11O, Supporting Information). Finally, to confirm enhanced Rilp recruitment by ML‐098 stimulation, immunoblotting of Rab7 and Rilp in Langendorff purified cardiomyocytes shows an increase in Rilp but not Rab7 protein levels (Figure 7O). Taken all together, our results suggested that activating Rab7 activity by enhancing Rilp protein levels can improve cardiac diastolic function in DbCM mice.

## 3 Discussion

T2DM‐induced dyslipidemia is a severe cause of cardiovascular diseases including coronary artery diseases (CAD) and DbCM.^[^ ^27^ ^]^ Unlike symptomatic CAD, DbCM are often asyptomatic and progressive, and like CAD, results in heart failure. Glucagon‐like peptide‐1 receptor agonists (GLP‐1RAs) and the sodium‐glucose cotransport inhibitors (SGLT2i) ^[^ ^28^ ^]^ are two recent new drugs that phycisians can offer to treat DM heart failure patients but lipid buildup and lipid toxicity problem remain unresolved. In DbCM patients, how their hearts deal with excessive lipid content and what protects them from lipid toxicity is undetermined. Here we show that lipophagy is significantly activated in DbCM and it helps to degrade overwhelmed lipid to protect cardiomyocytes from lipid toxicity. And this lipophagy is proceeded by autophagosome‐independent microlipophagy, which is a direct fusion between lysosome and LDs. Following this, using Rab7‐CKO mice and in vitro DbCM model, we demonstrated that this microlipophagy requires the activity of Rab7 and the recruitment of Rilp after phosphorylation of Rab7 in its Tyrosine 183 site. We discovered that the insufficient recruitment of Rilp in the long‐term lipid stimulation is responsible for lipid accumulation and toxicity in DbCM. Activating Rilp recruitment by Rab7 activator ML‐098 injection on DbCM mice successfully restore cardiac dysfunction caused by lipid toxicity.

As autophagy can be recognized as macroautophagy, microautophagy and chaperon mediated autophagy, lipophagy can be either autophagosome‐dependent or independent.^[^ ^29^ ^]^ Macrolipophagy, in which LDs are engulfed by an LC3‐dependent autophagosome and then fused with lysosome, has been comprehensively studied.^[^ ^30^, ^31^ ^]^ Microlipophagy, on the other side of lipophagy, is a new terminology reported in recent years and is not fully understood. Zutphen et al. demonstrated in yeast that LDs can also be turned over by a process that morphologically resembles microautophagy ^[32]^ while Schulze et al. demonstrated presence of microlipophagy in hepatocytes.^[^ ^33^ ^]^ In diabetic hearts, whether lipophagy exist, regardless of its form, is even controversial. Absence of double membrane bound LDs and LC3/LDs colocalization suggests absence of lipophagy in the heart ^[^ ^16^ ^]^; colocalization of LDs with lysosome marker Lamp1 suggests otherwise.^[^ ^17^ ^]^ Here we provide TEM evidence of the presence of lysosome‐LDs fusion and that in diabetic cardiomyocytes, lipophagy proceeded by autophagosome independent microlipophagy.

Some isoforms of autophagy can be measured by certain protein markers, for example, mitophagy can be determined by measuring PINK1/Parkin or others.^[^ ^34^ ^]^ Currently there is no consensus regarding molecular markers for microlipophagy and lipophagy.^[^ ^35^ ^]^ Recently, ORP8 has been demonstrated to be a marker for macrolipophagy ^[^ ^36^ ^]^ while Spartin has been suggested to mediate selective autophagy of LDs.^[^ ^37^ ^]^ Rab7 GTPase is previously recognized as a protein that mediated membrane trafficking and has been shown to regulate lipophagy in hepatocytes,^[^ ^19^ ^]^ mitochondria‐lysosome fusion,^[^ ^20^ ^]^ and mitochondria clearance through extracellular vesicle secretion (EVs).^[^ ^38^ ^]^ Our work here adds another role of Rab7 in regulating to lipophagy – actived Rab7 GTPase recruits its downstream effector Rilp, perhaps the rate limiting step, to mediate the fusion between LDs and lysosome. Molecularly, this Rilp recruitment requires phosphorylation of Tyrosine 183 site on Rab7. Our results provide a new foundation for targeting macrolipophagy in DbCM through the regulation of Rab7 and offer new molecular markers for validating lipophagy in cardiac and other systems.

It is clear that our work is not without limitations. First, our experiment explored multiple lipophagy pathways in diabetic cardiomyocytes but did not focus on lipophagy initiation, maturation, and consequence. Second, the negative feedback loop that turns off Rab7 GTPase after Rilp recruitment remains elusive. Last, ML‐098 pharmacokinetic data will need to be evaluated before considering translating into the clinics.

## 4 Conclusion

Here we identified that, in the context of DbCM lipid overload, microlipophagy, which significantly differs from traditional macrolipophagy, is activated to help degrade excessive LDs by direct fusion with lysosome. This microlipophagy required Rab7 GTPase activation, by Tyr183 phosphorylation, to recruit downstream Rilp. Prolonged lipid stimulation in DbCM results in insufficient Rilp recruitment but can be rescued with Rab7 activator ML‐098. Our work has broadened our understanding of lipophagy in diabetic cardiomyocytes and provides support for targeting Rab7 in treating lipid toxicity in DbCM.

## 5 Experimental Section

### Human Heart Biopsy

All human cardiac tissues used in this study were collected with informed written consent and approved by the Ethics Review Committee at the Ninth People's Hospital, Shanghai Jiaotong University School of Medicine, China (SH9H‐2020‐TK238‐1). All procedures performed were in accordance with the 1964 Helsinki Declaration and its later amendments or comparable ethical standards. The general clinical characteristics of patients are summarized in Table S2 (Supporting Information).

### Experimental Animals

The Animal Experiment Ethics Committee of Shanghai Ninth People's Hospital approved all animal experiments performed in this study (SH9H‐2020‐A231‐1). Animals were euthanized by cervical dislocation after deep isoflurane (5%) anesthesia and death was confirmed by exsanguination.

Wild type C57BL/6J adult mice were obtained from Shanghai Jihui Laboratory Animal Care Co.,Ltd. Myocardial Rab7 knockout (Rab7‐CKO) mice were generated by crossing tamoxifen‐inducible Myh6‐Cre^ERT2^ animals with Rab7^flox/flox^ animals (Shanghai Model Organisms Center, Inc., Shanghai, China). Two weeks before induction of DbCM, tamoxifen injection (MedChemExpress, 100 mg k^−1^g/day, once a day for 5 consecutive days) were operated intraperitoneally (*i.p*.) to induce cre recombinase activity. PCR was used to confirm genotype. All animals were housed under a light/dark cycle of 12:12 h (lights on at 6:00) at 22±2 °C. Mice received a standard chow diet (3.601 kcal/g; 9.4% fat, 14.7% proteins, 75.9% carbohydrates) and water was available ad libitum.

Type 2 diabetic mice were established by subjecting the 7‐weeks old animals to a 3‐month 60% high‐fat diet (5.128 kcal g^−1^; 60% fat, 20% protein, and 20% carbohydrates) followed by a 5‐day continuous *i.p*. injection of streptozocin (STZ; 50 mg k^−1^g, Sigma–Aldrich). Fed mice with glucose level exceeding 16.7 mmol l^−1^ were considered successful DbCM. Standard and high‐fat chow were obtained from Jiangsu Xietong Pharmaceutical Co., Ltd. For ML‐098 injection, ML‐098 (MedChemExpress, #HY‐19800, 1 mg k^−1^g/day, once a day for five consecutive days a week, injection for two weeks) were dissolved in DMSO and then diluted with corn oil and administered by *i.p*. injection.^[^ ^39^ ^]^

### Echocardiography and Doppler Imaging

For measuring cardiac function, transthoracic echocardiography was performed using a Visual Sonics Vevo 3100 system equipped with an MS400 transducer (FUJIFILM Visual Sonics). 2% isoflurane was used to induce anesthesia and then 0.5% to 1.0% isoflurane was designated to maintain a heart rate ranging from 425 to 475 beats per minute to stably obtain parameters. Systolic function was measured at the midventricular long‐axis using M‐mode scanning. To assess diastolic functions, pulsed‐wave tissue Doppler of the mitral valve was operated in the view of the apical four‐chamber while maintaining the heart rate at the range of 340 to 390 beats per minute. The following parameters were collected and analyzed: Left ventricular (LV) ejection fraction (LVEF), LV end‐diastolic anterior wall (LVAW:d), LV end‐diastolic posterior wall (LVPW:d), LV global longitudinal strain (GLS), peak Doppler blood inflow velocity across the mitral valve during early diastole (E), peak Doppler blood inflow velocity across the mitral valve during late diastole (A), peak tissue Doppler of myocardial relaxation velocity at the mitral valve annulus during early diastole (E’).

### Blood Glucose Measurement, Glucose Tolerance Test, and Insulin Tolerance Test

Blood glucose was measured with tail vein sampling by Accu‐Check (Roche). Before the injection of STZ, a glucose tolerance test (GTT) was performed by *i.p*. injecting α‐d‐glucose (#G8270, Sigma‐Aldrich, 2 g kg^−1^ body weight) after 6 h of fasting. Blood glucose levels were measured at 0, 15, 30, 60, 90, and 120 min after glucose injection and were recorded for analysis. After the injection of STZ, insulin tolerance test (ITT) was performed after 3 h of fasting by *i.p*. injecting acetic acid (adjusting pH to 2–3, final concentration 2 mg ml^−1^) dissolved, saline diluted (at 1:1000 to 1U/kg body weight) insulin (Aladdin Scientific Corp, #1 302 196). Blood glucose levels were measured at 0, 15, 30, 60, 90, and 120 min after insulin injection and were recorded for analysis.

### Histology and Oil‐Red Staining

For determining cardiac fibrosis, hearts from scarified mice were surgically excised and fixed in 4% paraformaldehyde in Phosphate Buffered Saline (PBS; #G0002, Servicebio Biotech, China) overnight at 4 °C. After embedding with paraffin, tissues were sectioned (5 µm) and Masson's trichrome staining was performed by manufacturer's instructions (G1343, Solarbio, China). Degree of fibrosis (%) was quantified using ImageJ software version 2.0.

For staining lipid accumulation, 10 µm cryosections of sacrificed mice heart was stained with commercial Oil‐Red staining kit (C0158S, Beyotime Biotechnology, China). Micrographs were captured by microscope (IX83, OLYMPUS, Japan) and quantified by ImageJ software version 2.0.

### Cardiomyocyte Isolation

Adult mouse ventricular myocytes (AMVMs) were isolated using a Langendorff perfusion system. In brief, mice were anesthetized with 5% isoflurane and sacrificed by cervical dislocation. Murine hearts were isolated and immediately placed on ice‐cold cardiomyocyte isolation buffer (CIB; containing 120 mm NaCl, 5.4 mm KCl, 0.5 mm MgSO_4_, 0.33 mm NaH_2_PO_4_, 25 mm NaHCO_3_, 22 mm glucose, 25 mm HEPES, 10 mm BDM and 30 mm taurine) before cannulated for collagenase digestion (1 mg mL^−1^ Type II Collagenase, 0.6 mg mL^−1^ Type IV Collagenase for 15 min at 37 °C). Digested tissues were dissociated by tweezers and then transferred into 50 mL^−1^ centrifuge tube and shaked at 37 °C (100 rpm) for further digestion. The digestion mixture was triturated using a pipette and the digestion reaction was arrested by the addition of Minimum Essential Medium Eagle (MEM; #M0518, Sigma–Aldrich) supplemented with 10% bovine serum albumin (BSA; Sigma–Aldrich). The digestion mixture was filtered and AMVMs were spun down at 500 rpm. The supernatant (containing fibroblasts and endothelial cells) was discarded, AMVMs were resuspended in fresh medium, and calcium was reintroduced in 4 steps, ranging from 0 to 900 µM.

Neonatal mouse cardiomyocytes (NMCMs) were isolated from C57BL/6 J pups (within 36 h). In brief, hearts were surgically isolated and immediately digested in Trypsin–EDTA Solution (C0201, Beyotime Biotechnology, China). After neutralization with DMEM buffer (12430054, Gibco, USA, supplemented with 10% fetal bovine serum (10270‐106, Gibco, USA)), cell suspensions were centrifuged at 1000 rpm for 5 min and resuspended in DMEM buffer. The cells were filtered through a 100 µm mesh filter, and inoculated into culture dishes, and incubated at 37 °C in a humidified incubator containing 95% air and 5% CO_2_ for 1 h. The upper cell suspension, which contains the unattached NMCMs, was collected and inoculated into new culture dishes while the adherent cardiac fibroblasts were disposed. NMCMs were allowed to adhere for 3 days before washed by DMEM to remove non‐adherent blood cells. Experiments or drug incubations were carried out on day 4 NMCMs.

HL‐1 cardiac muscle cell line and HEK 293T cell line were acquired from Shanghai Zhong Qiao Xin Zhou Biotechnology Co.,Ltd.

### Myocardial Contractility Assay

AMVMs were seeded onto glass bottom dishes pretreated with laminin (Sigma–Aldrich, L2020). After incubation in DMEM buffer for 30 min, a field stimulator was immersed into the DMEM buffer to electrically stimulate AMVMs at 1 Hz and contractility was recorded using an IonOptix HTC system (IonOptix).

96‐well plates containing NMCMs (seeding at a density of 50–60% per field) were placed on a live‐cell working station (37 °C, 5% CO_2_) to maintain cellular functions. Briefly, NMCM contraction videos were captured on a IX83 microscope (OLYMPUS, Japan) at 40x DIC mode to acquire a 10 s video with 50 frames per second. The exported videos were calculated for pixel deviation and contraction velocity using MATLAB (MathWorks, USA) as previously described.^[^ ^40^ ^]^

### Cell Treatment and Transfection

In vitro DbCM model was established by incubating cells with high glucose and palmitate (HGPA) solution as previously described.^[^ ^21^, ^22^ ^]^ The HGPA solution is prepared as follows. Palmitate acid (P0050, Sigma–Aldrich) was dissolved in ethanol at 0.1 mol L^−1^ and then bound to 10% low‐fatty acid Bovine Serum Albumin (BSA) (B2064, Sigma‐Aldrich, dissolved in ddH_2_O) to a concentration of 10 mmol L^−1^. After mixing with high glucose (33 mmol L^−1^) DMEM buffer to a final concentration of 0.5 mmol L^−1^, a solution was filtered by a 0.22 µm filter and stored at 4 °C prior to use. The control solution (Ctrl) was prepared by dissolving mannitol (M4125, Sigma–Aldrich) into DMEM buffer to a final concentration of 33 mmol L^−1^, to match the osmotic pressure of the HGPA buffer, before mixing with 10% filtered BSA solution.

For transfection of siRNA, HL‐1 cell were seeded in a six‐well plate and transfected with siRilp (Genomeditech Co., Ltd. (Shanghai, China)) and si‐control according to the manufacturer's instructions. For transfection of plasmid, HEK 293T was transfected with FLAG‐Rab7 (WT), FLAG‐Rab7 (S17A), FLAG‐Rab7 (S72A), FLAG‐Rab7 (Y183A) and HA‐Rilp plasmids (Genomeditech Co., Ltd. (Shanghai, China)) and vector control according to the manufacture instructions. For transfection of RFP‐GFP‐LC3 lentivirus, HL‐1 cells were seeded in a six‐well plate or 96‐well plates and transfected with RFP‐GFP‐LC3 (Genomeditech Co., Ltd. (Shanghai, China)) following the manufacture's protocol. For transfection of lentivirus, NMCMs or HL‐1 cells were seed in 6 cm dishes (for immunoprecipitation), 6‐well plates (for immunoblots) or 96‐well plates (for immunoflurorences) before transfecting with GST‐Rilp or Rab7^Y183A^ lentivirus following protocols from Genomeditech Co., Ltd. (Shanghai, China).

The following reagents were used for treatments: Rapamycin (MedChemExpress, a final concentration of 50 µm), 3‐MA (MedChemExpress, a final concentration of 5 mm), CID‐1067700 (MedChemExpress, a final concentration of 40 µm), ML‐098 (MedChemExpress, a final concentration of 0.5 µm).

### Real‐Time Quantitative Polymerase Chain Reaction

RNA was extracted from murine hearts, HL‐1 cells, and NMCMs using the Trizol reagent (R0011, Beyotime Biotechnology, China) per manufacturer's instructions and reverse‐transcribed into cDNA using the PrimeScript RT reagent Kit (#RR037B, TAKARA). The cDNA was then amplified using SYBR Green qPCR Master Mix (#B21703, Bimake, USA or #Q711, Vazyme, China), and Real‐time qPCR reactions were performed in triplicates using an Applied Biosystems 6Flex system (ABI, USA). The fold increase of mRNA expression was calculated using the 2‐ΔΔCq method. Forward and reverse primer sequences are listed in Table S3 (Supporting Information).

### RNA‐Seq Analysis

Total RNAs were extracted from HL‐1 cells cultured under HGPA or control medium using the Trizol reagent (R0011, Beyotime Biotechnology, China) according to the manufacturer's protocol. RNA purity and quantification were evaluated using a NanoDrop 2000 spectrophotometer (Thermo Scientific, USA) while RNA integrity was evaluated using an Agilent 2100 Bioanalyzer (Agilent Technologies, Santa Clara, CA, USA). cDNA libraries were constructed using VAHTS Universal V6 RNA‐seq Library Prep Kit according to the manufacturer's instructions. The libraries were sequenced on an Illumina Novaseq 6000 platform and 150 bp paired‐end reads were generated. Sequencing and standard analyses were performed by OE Biotech Co., Ltd. (Shanghai, China).

### Immunoblots and Immunoprecipitation

For immunoblots, proteins were extracted from Langendroff AMVMs and NMCMs following the procedures below. Langendroff AMVMs and NMCMs were grinded for 2 min before lysed by radioimmunoprecipitation (RIPA) buffer (P0013B, Beyotime Biotechnology, China) complemented with cocktail protease inhibitor (P1045, Beyotime Biotechnology, China) at 4 °C for 30 min. The lysis buffer was centrifugated at 12,000 rpm for 15 min at 4 °C, and the concentration of the supernatant protein was determined using the bicinchoninic acid (BCA) method (P0012, Beyotime Biotechnology, China). Proteins (20 µg) were size‐fractionated by sodium dodecyl sulphate polyacrylamide gel electrophoresis (SDS‐PAGE) and separated by 7.5–15% gradient gels (Bio‐Rad, USA) before being transferred onto Immobilon polyvinylidene difluoride PVDF (IPVH00010, Millipore, Germany) membranes. The membranes were blocked with 5% DifcoTM Skim Milk (BD10610, BD Biosciences, USA) for 2 h at room temperature and incubated with primary antibodies at 4 °C overnight. The membranes were washed and incubated with secondary antibody (anti‐rabbit IgG, #5151S, Cell signaling technology, 1:100 000; Anti‐mouse IgG, #5257S, Cell signaling technology, 1:100000) for 60 min at 37 °C. Results were visualized by Odyssey Infrared Imaging System (LICOR, USA) and analyzed by ImageJ software version 2.0.

For immunoprecipitation, proteins were extracted from HL‐1 cell and HEK 293T cell. Briefly, cells after treatment were collected and then mixed with lysis buffer (P0013, Beyotime Biotechnology, China) containing cocktail protease inhibitor (P1045) before a brief sonication. The concentration of supernatant protein was determined using the bicinchoninic acid (BCA) method (P0012, Beyotime Biotechnology, China). Samples were incubated with antibody /anti‐GST beads /anti‐FLAG beads to be cultured at 4 °C overnight. The isotype IgG antibody (#B900620, Proteintech or #2729S Cell Signaling Technology) was used as a negative control. For the binding of the primary antibody, samples were incubated with Protein A/G magnetic beads (B23201, Bimake, USA) overnight again. The precipitated beads were washed gently for 3 times with ice‐cold lysis buffer and proteins were eluted by sodium dodecyl sulphate polyacrylamide gel electrophoresis (SDS‐PAGE) by incubating at 95 °C for 10 min. Eluted protein levels were assayed by immunoblotting. Results were quantified by ImageJ software version 2.0 and relative intensity of each band is given in Figure S12 (Supporting Information).

All antibodies were listed as follows: LC3 (Cell Signaling Technology, #3868S, 1:1000), Rab7 (Cell Signaling Technology, #95746S, 1:1000), GAPDH (Proteintech, #60004‐1, 1:100 000), GST (Yeasen Biotechnology, 30902ES, 1:1000), Ulk1 antibody (Proteintech, 20986‐1‐AP, 1:1000), Atg5 antibody (Proteintech, 10181‐2‐AP, 1:1000), Rilp antibody (Invitrogen, PA5‐34357, 1:1000), Ubiquitin antibody (Cell Signaling Technology, #3936S, 1:1000), Atf6 (Proteintech, #24169‐1‐AP, 1:2000), Ire1α (Cell Signaling Technology, #3294S, 1:1000), Phosphotyrosine (pTyr, Santa Cruz, #sc‐7020).

### Immunofluorescence Staining

Sacrificed mice hearts were embedded with optimal cutting temperature compound (OCT, #4583, SAKURA Tissue‐Tek O.C.T.). Cryosections (10 µm) were rinsed with PBS, and then fixed with 4% paraformaldehyde at room temperature for 20 min. After 3 washes, the cryosection was perfused by Triton X‐100 (P0256, Beyotime Biotechnology, China) for 10 mins and then blocked by 5% goat serum (C0265, Beyotime Biotechnology, China) for 1 h at 37 °C, and incubated with primary antibodies or Lipi‐Red probe (#LD03 DOJINDO LABORATORIES), overnight at 4 °C. Slides were washed three times and incubated with Alexa Fluro488 or Alexa Fluro647 conjugated IgG (Cell Signaling Technology, USA, 1:1000 diluted in PBS buffer) for 1 h at room temperature. Nuclei were stained using 4,6‐diamidino‐2‐phenylindole (DAPI, P0131, Beyotime Biotechnology, China) for 5 min at room temperature. Micrographs were acquired using a Zeiss LSM 880 upright confocal fluorescence microscope. Colocalization rate (Pearson's R) and Lipi‐red staining area (%) were calculated by Image J and Fiji.

For live‐cell tracing of lipophagy, NMCMs were seeded on 96‐well plates (an estimated 5×10^4^ cells per field) and stained with Lysotracker (#40738ES50, Yeasen Biotechnology), Lipi‐Red probe (DOJINDO LABORATORIES, #LD03) and Hoechst 33 342 (C1022, Beyotime Biotechnology, China) before imaging on a Zeiss LSM 880 upright confocal fluorescence microscope with live‐cell working station (37 °C, 5% CO_2_). Colocalization rate (Pearson's R) and Lipi‐Red staining area (%) were calculated by Image J and Fiji.

For live‐cell tracing of cellular reactive oxygen species (ROS), NMCMs or AMVMs were seeded on 96‐well plates (an estimated 5×10^4^ cells per field) and stained with DCFH‐DA Dye (DOJINDO LABORATORIES, #R252) according to manufactures’ protocol. Images were then acquired by Operetta CLS High Content Imaging System (Perkin Elmer, USA).

The following reagents were used in the immunofluorescence staining: Lamp1 (Santa Cruz Biotechnology, #H4A3), Lipi‐Red (DOJINDO LABORATORIES, #LD03), LC3 (Cell Signaling Technology, #3868S), Rab7 (Cell Signaling Technology, #95746S), Ulk1 (Proteintech, 20986‐1‐AP, 1:1000), Atg5 (Proteintech, 10181‐2‐AP, 1:1000) and DCFH‐DA Dye (DOJINDO LABORATORIES, #R252).

### Transmission Electron Microscopy (TEM)

Sacrificed mice hearts were immediately fixed in 2.5% glutaraldehyde overnight at 4 °C. Samples were washed with 0.1 m cacodylate trihydrate buffer for 3 times and post‐fixed with 1% osmium tetroxide for 1 h. After 3 washes with PB Buffer, samples were dehydrated according to manufactures’ protocol. Sections were observed in a Philips CM‐10 TEM (FEI Italia, 20 122 Milan, Italy).

### Enzyme‐Linked Immunosorbent Assay (ELISA)

To measure mouse plasma insulin level, blood sample from sacrificed mice were collected into sodium citrate‐soaked centrifuge Tubes for 20 min before being centrifuged at 1000 rpm for 15 min at 4 °C. Supernatants were collected for Enzyme‐linked immunosorbent assay (ELISA) following protocols from Mouse insulin ELISA KIT (SEKM‐0141, Solarbio, China).

### Triglyceride (TG) Content Assay

For determining triglyceride content, murine hearts or NMCMs were treated with isopropanol and then centrifuged at 12000 g, 4 °C for 5 min. The triglyceride were then measured following the protocols from Amplex Red Triglyceride Assay Kit (S0219S, Beyotime Biotechnology, China).

### Statistical Analysis

Statistical analysis was performed and graphed by using GraphPad Prism software (Version 9.1.1). All arithmetic data were presented as mean ± SD at least three independent experiments. The Student's *t*‐test was used to analyze the differences between 2 groups. All *p* value < 0.05 was considered statistically significant.

## Conflict of Interest

The authors declare no conflict of interest.

## Author Contributions

JK, JZ, CW, ACYC and JG developed the study concept and experimental design; JK, JP, HL and SH conducted the experiments and analyzed the results; All authors read and approved the final manuscript.

## Supporting information

Supporting Information

## Data Availability

The data that support the findings of this study are available from the corresponding author upon reasonable request.
