# Supplementary material for: Targeting Rab7‐Rilp Mediated Microlipophagy Alleviates Lipid Toxicity in Diabetic Cardiomyopathy
Source: Adv Sci (Weinh). 2024 Jun 5;11(29):2401676. doi: 10.1002/advs.202401676 (PMC11304244; doi:10.1002/advs.202401676)
Supplement: Supplementary file 1 — Supporting Information [file ADVS-11-2401676-s001.docx]

Supporting Information

Targeting Rab7-Rilp mediated microlipophagy alleviates lipid toxicity in diabetic cardiomyopathy

*Jiahan Ke and Jun Gu**

# *Supplementary* Tables

***Table S1 Genes expression***

|  |  |  | Expression | | | | | |
| --- | --- | --- | --- | --- | --- | --- | --- | --- |
|  | p-Value | q-Value | Ctrl1 | Ctrl2 | Ctrl3 | HGPA1 | HGPA2 | HGPA3 |
| Rab7 | 2.81E-10 | 8.08E-09 | 137.187 | 140.779 | 140.197 | 165.467 | 163.592 | 168.272 |
| Kat5 | 0.02565 | 0.12018 | 13.8265 | 14.1283 | 14.1283 | 15.4834 | 16.0722 | 14.5979 |
| Atg2a | 0.12864 | 0.36692 | 6.898 | 7.046 | 7.2437 | 7.5797 | 7.9191 | 7.4563 |
| Prkaa1 | 0.05853 | 0.21715 | 25.546 | 24.4829 | 23.3049 | 22.2848 | 20.7851 | 24.0916 |
| Atg2b | 0.43893 | 0.72149 | 7.3053 | 7.1759 | 6.3893 | 6.5039 | 7.137 | 6.3591 |
| Prkaa2 | 0.9755 | 0.99102 | 0.3754 | 0.1505 | 0.2043 | 0.3208 | 0.218 | 0.1615 |

***Table S2 Patients characteristics***

|  | age | sex | Diagnosis | Sampling location |
| --- | --- | --- | --- | --- |
| patient 1 | 66 | male | aortic stenosis; aortic insufficiency | Left auricle |
| patient 2 | 63 | male | Sinus tachycardia | Left auricle |
| patient 3 | 67 | male | type 2 diabetes; cardiac dysfunction; primary hypertension | Left auricle |

***Table S3 Primers used in experiment***

| m-Cd36-F | 5’- GGAGCCATCTTTGAGCCTTCA-3’ |
| --- | --- |
| m-Cd36-R | 5’-GAACCAAACTGAGGAATGGATCT-3’ |
| m-Fabp3-F | 5’- ACCTGGAAGCTAGTGGACAG-3’ |
| m-Fabp3-R | 5’- TGATGGTAGTAGGCTTGGTCAT-3’ |
| m-Hsl-F | 5’- TGGCACACCATTTTGACCTG-3’ |
| m-Hsl-R | 5’- TTGCGGTTAGAAGCCACATAG-3’ |
| m-Atgl-F | 5’- GGTCCTCCGAGAGATGTGC-3’ |
| m-Atgl-R | 5’-TGGTTCAGTAGGCCATTCCTC-3’ |
| m-Rilp-F | 5’- TTCCAGCGAGAGCTGCTCAC-3’ |
| m-Rilp-R | 5’- CATCCTCACTGCTCTCTGC-3’ |
| m-Gapdh-F | 5’-AGGTCGGTGTGAACGGATTTG-3’ |
| m-Gapdh-R | 5’-GGGGTCGTTGATGGCAACA-3’ |
| m-Tnf-F | 5’-TCGTAGCAAACCACCAAGTG-3’ |
| m-Tnf-R | 5’-TTGTCCCTTGAAGAGAACCTG-3’ |
| m-Il6-F | 5’-TGCAAGAGACTTCCATCCAG-3’ |
| m-Il6-R | 5’-ATTTCCACGATTTCCCAGAG-3’ |
| m-Col1a1-F | 5’-TTCTCCTGGCAAAGACGGAC-3’ |
| m-Col1a1-R | 5’-CGGCCACCATCTTGAGACTT-3’ |
| m-Col3a1-F | 5’-GCGAGCGGCTGAGTTTTATG-3’ |
| m-Col3a1-R | 5’-GCAGCTCAGAGTAGCACCAT-3’ |
| m-Nppa-F | 5’-GCTTCCAGGCCATATTGGAG-3’ |
| m-Nppa-R | 5’-GGGGGCATGACCTCATCTT-3’ |
| m-Nppb-F | 5’-GAGGTCACTCCTATCCTCTGG-3’ |
| m-Nppb-R | 5’-GCCATTTCCTCCGACTTTTCTC-3’ |
| m-Myh7-F | 5’-ACTGTCAACACTAAGAGGGTCA-3’ |
| m-Myh7-R | 5’-TTGGATGATTTGATCTTCCAGGG-3’ |

# *Supplementary Figures*

***Figure S1***


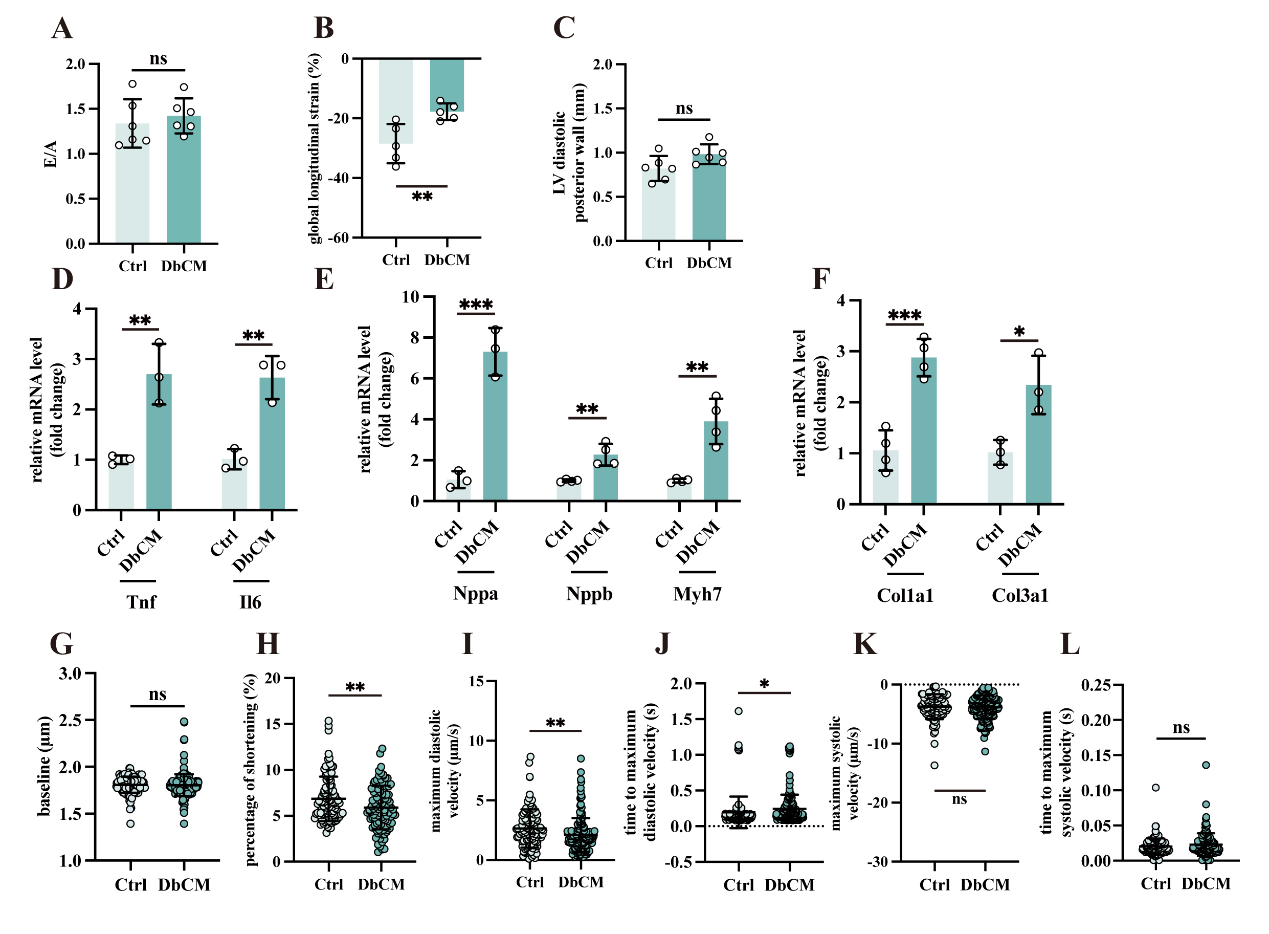


Figure S1. Presence of lipid toxicity in DbCM. **A** Evaluation of E/A (n=6 per group). **B** Evaluation of global longitudinal strain (GLS) (n=5 per group). **C** Analysis on thickness of left ventricular diastolic posterior wall (LVPW:d) (n=6 per group). **D** Relative mRNA expression of Tnf and Il6 in DbCM mice hearts (n=3 per group). **E** Relative mRNA expression of Nppa, Nppb and Myh7 in DbCM mice hearts (n=3-4 per group). **F** Relative mRNA expression of Col1a1 and Col3a1 in DbCM mice hearts (n=3-4 per group). **G** Evaluation of baseline sarcomere length in Langendorff-isolated AMVMs. (at least 90 cells from 3 individual mice per group). **H** Evaluation on percentage of shortening in Langendorff-isolated AMVMs. (at least 90 cells from 3 individual mice per group). **I** Evaluation on maximum diastolic velocity in Langendorff-isolated AMVMs. (at least 90 cells from 3 individual mice per group). **J** Evaluation of time to maximum diastolic velocity in Langendorff-isolated AMVMs (at least 90 cells from 3 individual mice per group). **K** Evaluation on maximum systolic velocity in Langendorff-isolated AMVMs (at least 90 cells from 3 individual mice per group). **L** Evaluation of time to maximum systolic velocity in Langendorff-isolated AMVMs (at least 90 cells from 3 individual mice per group). The Student's t-test was used to analyze the differences between 2 groups and the data are expressed as mean ± SD, ns: no significance, *p < 0.05, **p < 0.01 and ***p < 0.001.

***Figure S2***


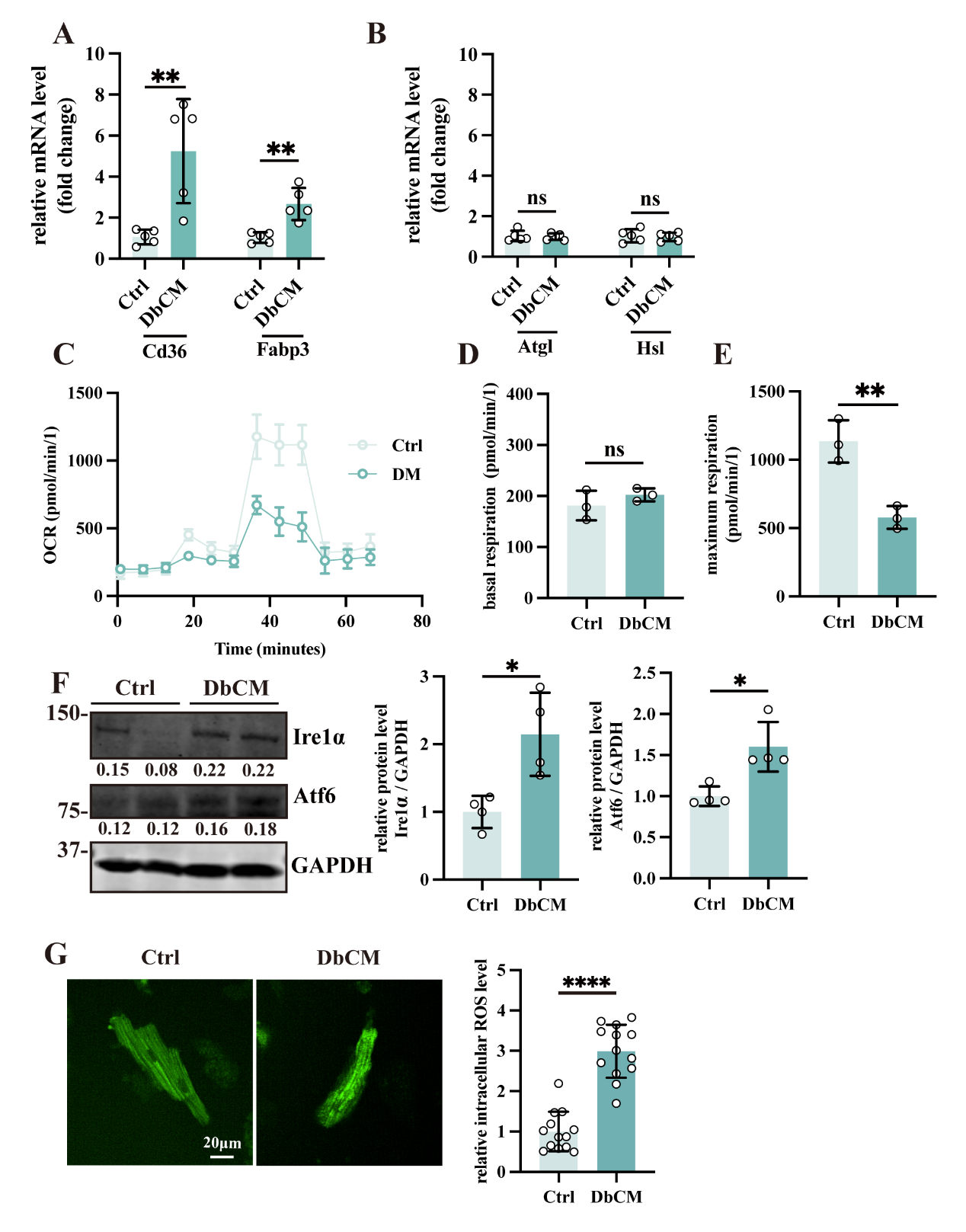


Figure S2. Altered lipid homeostasis and lipid toxicity in DbCM. **A** Relative mRNA expression of Cd36 and Fabp3 in DbCM mice heart (n=5 per group). **B** Relative mRNA expression of Atgl and Hsl in DbCM mice heart (n=5 per group). **C** Real-time oxygen consumption rates (OCR) and calculated **D** basal and **E** maximum respiration rates in isolated left ventricular cardiomyocytes (n = 3 per group). **F** Representative blots, relative intensity and quantitative analysis of immunoblots analysis of Ire1α and Atf6 in Langendroff AMVMs (n=4 per group). **G** Representative micrograph and quantitative analysis of intracellular reactive oxygen species (ROS) in Langendroff AMVMs (n=13 per group). The Student's t-test was used to analyze the differences between 2 groups and the data are expressed as mean ± SD, ns: no significance, *p < 0.05, **p < 0.01 and ****p < 0.0001..

***Figure S3***


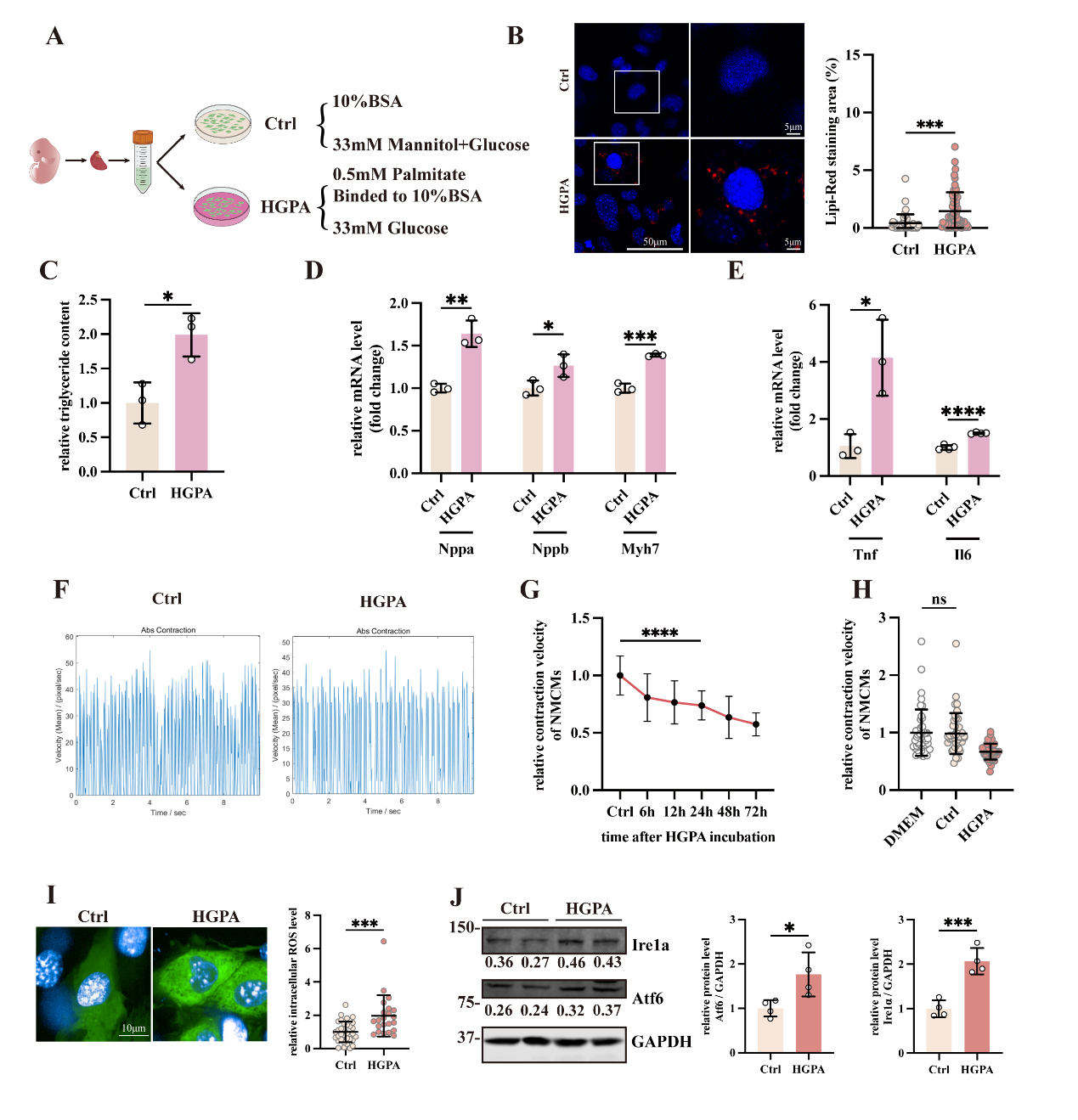


Figure S3. Establishment of in vitro DbCM model. **A** Experiment design for the establishment of in vitro DbCM model. **B** Representative micrographs of immunofluorescence staining of Lipi-Red to label LDs and quantification of Lipi-Red Staining Area (%) (at least 45 cells per group from 3 individual tests). **C** Relative triglyceride level in HGPA incubated NMCMs (n=3 per group). **D** Relative mRNA expression of Nppa, Nppb and Myh7 in HGPA incubated NMCMs (n=3 per group). **E** Relative mRNA expression of Tnf and Il6 in DbCM mice hearts (n=3 per group). **F** Representative contraction velocity figure in Ctrl and HGPA treated NMCMs. **G** Relative contraction velocity of HGPA incubated NMCMs (at least 30 cells per group from 3 individual tests). **H** Relative contraction velocity of normal DMEM media, control media (Ctrl) and HGPA incubated NMCMs (at least 40 cells from 3 individual tests per group). **I** Representative micrograph and quantitative analysis of intracellular reactive oxygen species (ROS, green) in HGPA treated NMCMs (at least 20 cells from 3 individual tests per group). Cell nuclei were labeled with Hoechst 33342 (blue). **J** Representative blots, relative intensity and quantitative analysis of immunoblots analysis of Ire1α and Atf6 in HGPA treated NMCMs (n=4 per group). The Student's t-test was used to analyze the differences between 2 groups and the data are expressed as mean ± SD, ns: no significance, *p < 0.05, **p < 0.01, ***p < 0.001 and ****p < 0.0001.

***Figure S4***


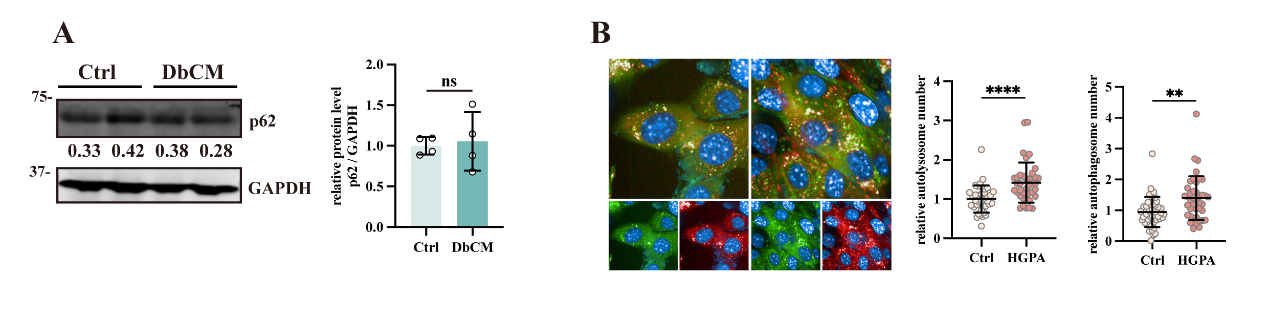


Figure S4. Autophagy flux increased in DbCM. **A** Representative blots, relative intensity and quantitative analysis of immunoblots analysis of p62 in Langendorff-isolated AMVMs (n=4 per group). **B** Representative micrograph and quantitative analysis of intracellular autolysosome (red signals) and autophagosome (yellow signals) after HL-1 cells were transfected with RFP-GFP-LC3 lentivirus (at least n=30 from 3 individual tests per group). Cell nuclei were labeled with Hoechst 33342. The Student's t-test was used to analyze the differences between 2 groups and the data are expressed as mean ± SD, ns: no significance, **p < 0.01, ****p < 0.0001.

***Figure S5***


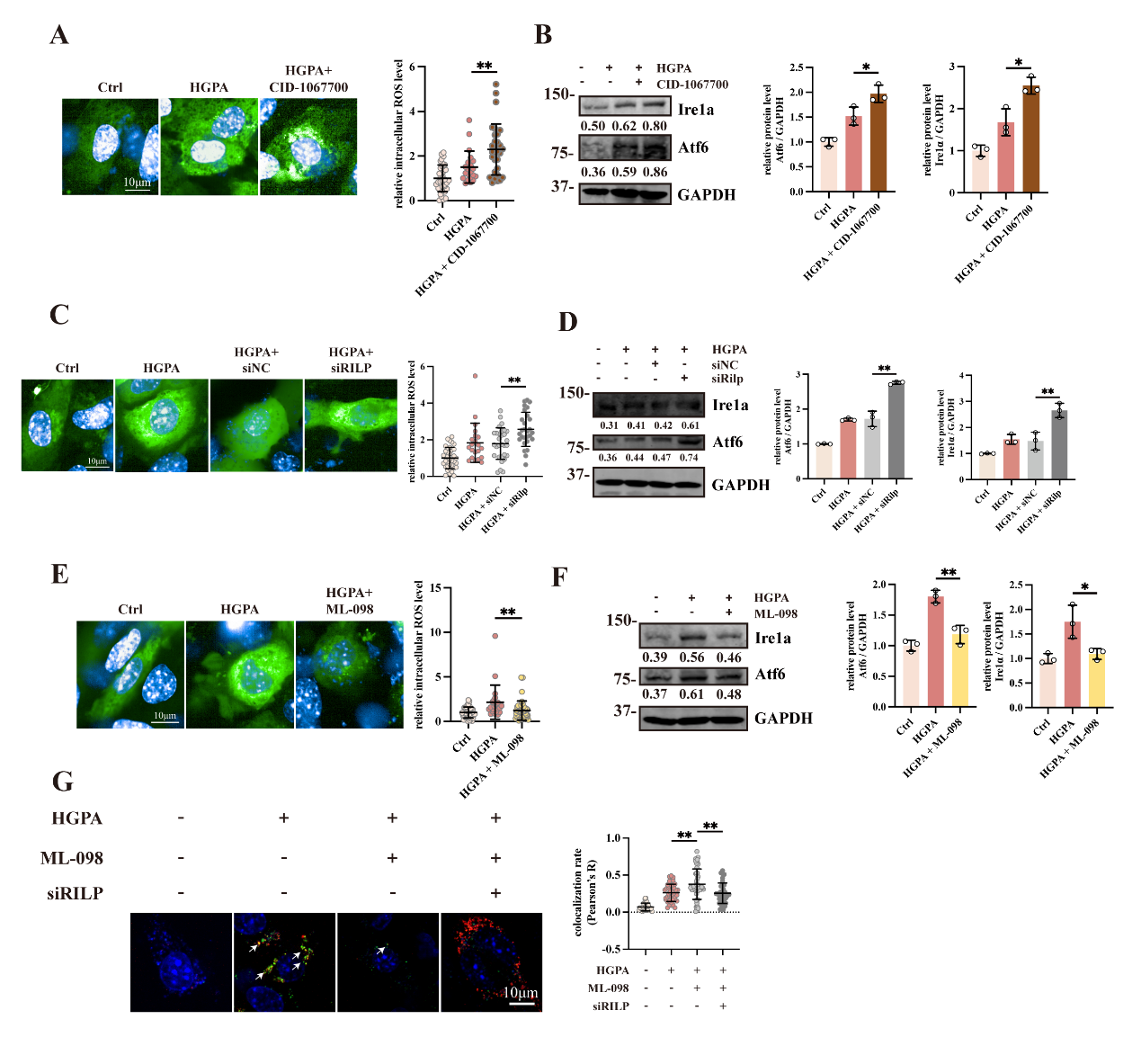


Figure S5. Regulating Rab7 influence lipophagy and lipid toxicity in DbCM. **A** Representative micrograph and quantitative analysis of intracellular reactive oxygen species (ROS, green) in Ctrl, HGPA and CID-1067700 treated NMCMs (at least 20 cells per group from 3 individual tests). Cell nuclei were labeled with Hoechst 33342 (blue). **B** Representative blots, relative intensity and quantitative analysis of immunoblots analysis of Ire1α and Atf6 in Ctrl, HGPA and CID-1067700 treated NMCMs (n=3 per group). **C** Representative micrograph and quantitative analysis of intracellular reactive oxygen species (ROS, green) in Ctrl, HGPA, siNC and siRILP treated NMCMs (at least 20 cells per group from 3 individual tests). Cell nuclei were labeled with Hoechst 33342 (blue). **D** Representative blots, relative intensity and quantitative analysis of immunoblots analysis of Ire1α and Atf6 in Ctrl, HGPA, siNC and siRILP treated NMCMs (n=3 per group). **E** Representative micrograph and quantitative analysis of intracellular reactive oxygen species (ROS, green) in Ctrl, HGPA and ML-098 treated NMCMs (at least 20 cells per group from 3 individual tests). Cell nuclei were labeled with Hoechst 33342 (blue). **F** Representative blots, relative intensity and quantitative analysis of immunoblots analysis of Ire1α and Atf6 in Ctrl, HGPA and ML-098 treated NMCMs (n=3 per group). **G** Live-cell confocal micrographs of NMCMs cells stained with Lipi-Red (red) to label LDs and Lysotracker (green) to label lysosome, and quantification of Lipi-Red colocalizing LysoTracker after they were incubated Ctrl, HGPA, and HGPA with ML-098 treated with or without siRilp. Cell nuclei were labeled with Hoechst 33342 (blue). Results were presented as Pearson’ R (at least 30 cells per group from 3 individual tests). The Student's t-test was used to analyze the differences between 2 groups and the data are expressed as mean ± SD, ns: no significance, *p < 0.05, **p < 0.01.

***Figure S6***


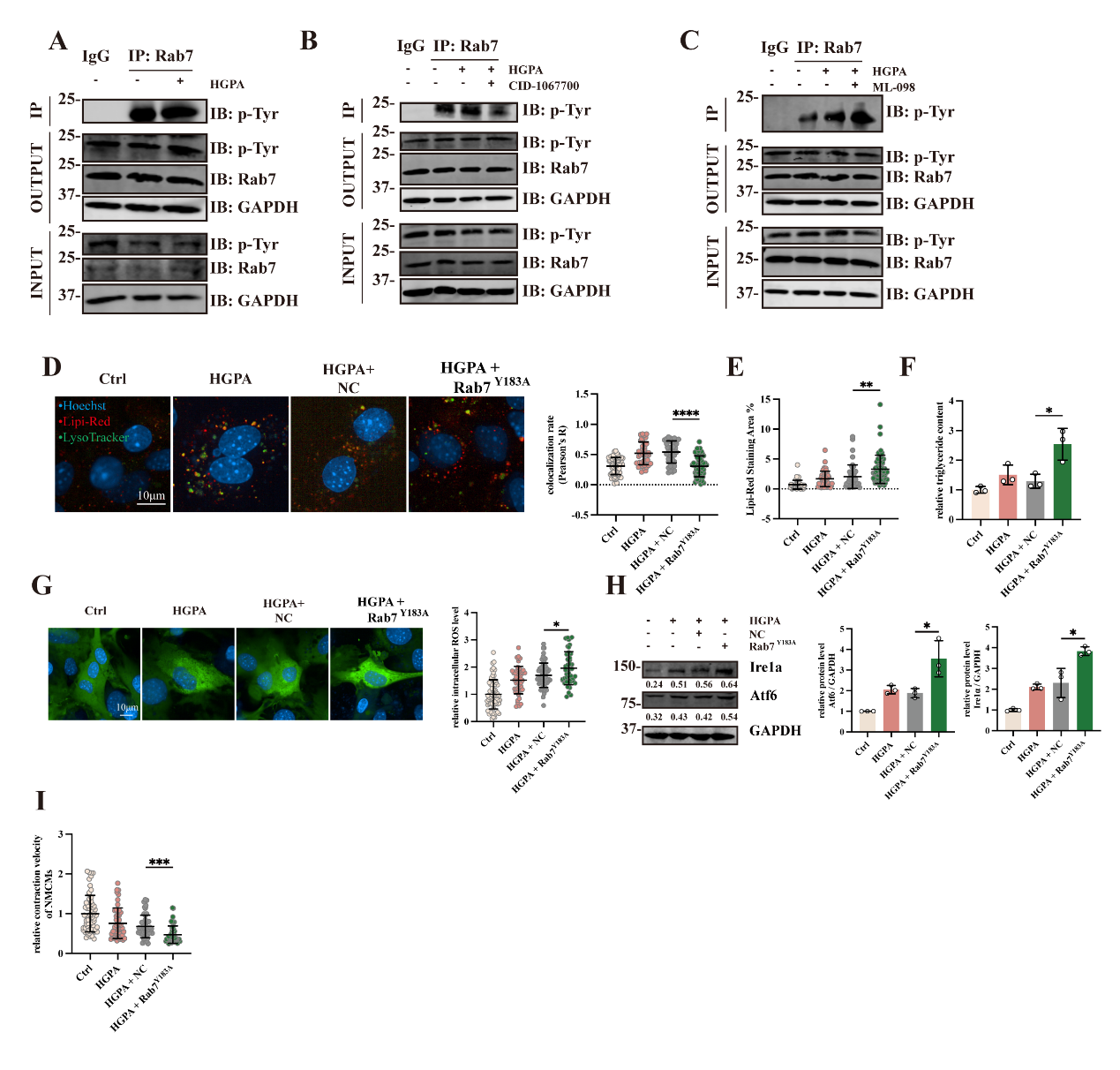


Figure S6. Rab7 tyrosine phosphorylation is required for DbCM microlipophagy. **A** Representative blot of Rab7 immunoprecipitated p-Tyr (phosphotyrosine) after treatment of HGPA on HL-1 cells. **B** Representative blot of Rab7 immunoprecipitated p-Tyr (phosphotyrosine) after treatment of Ctrl, HGPA and CID-1067700 on HL-1 cells. **C** Representative blot of Rab7 immunoprecipitated p-Tyr (phosphotyrosine) after treatment of Ctrl, HGPA and ML-098 on HL-1 cells. **D** Live-cell confocal micrographs of NMCMs cells stained with Lipi-Red (red) to label LDs and Lysotracker (green) to label lysosome and quantification of Lipi-Red colocalizing LysoTracker after they were incubated Ctrl, HGPA, and HGPA treated with or without Rab7^Y183A^. Cell nuclei were labeled with Hoechst 33342 (blue). Results were presented as Pearson’ R (at least 40 cells per group from 3 individual tests). **E** Quantification of Lipi-Red Staining Area (%) in Ctrl, HGPA, HGPA with or without Rab7^Y183A^ transfected NMCMs (at least 45 cells per group from 3 individual tests). **F** Quantification of relative triglyceride content in NMCMs after Rab7^Y183A^ transfection (n=3 per group). **G** Representative micrograph and quantitative analysis of intracellular reactive oxygen species (ROS, green) in Ctrl, HGPA, HGPA with or without Rab7^Y183A^ transfected NMCMs (at least 40 cells per group from 3 individual tests). Cell nuclei were labeled with Hoechst 33342 (blue). **H** Representative blots, relative intensity and quantitative analysis of immunoblots analysis of Ire1α and Atf6 in Ctrl, HGPA, HGPA with or without Rab7^Y183A^ transfected NMCMs (n=3 per group). **I** Relative contraction velocity of Ctrl, HGPA, HGPA with or without Rab7^Y183A^ transfected NMCMs (at least 45 cells per group from 3 individual tests). The Student's t-test was used to analyze the differences between 2 groups and the data are expressed as mean ± SD, *p < 0.05, **p < 0.01, ***p < 0.001 and ****p < 0.0001.

***Figure S7***


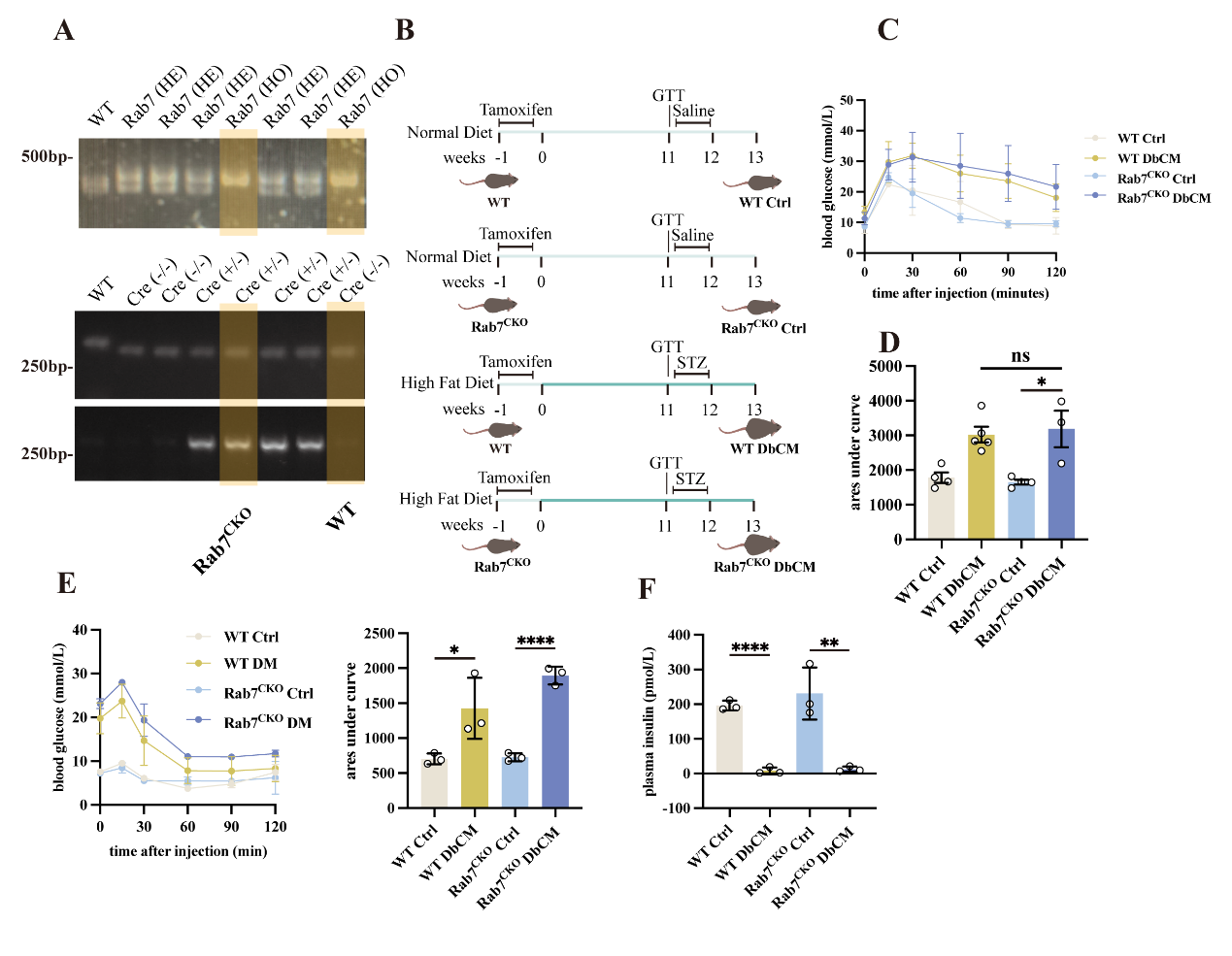


Figure S7. Establishment and Rab7-CKO (cardiac specific conditional knockout) mice. **A** Representative PCR micrograph of Rab7-CKO mice genotyping. **B** A flowchart describing the establishment of DbCM mice model in Rab7-CKO mice and wild type mice. **C** Changes in blood glucose level measured after injection of α-d-glucose on mice. **D** Quantification of areas under curve on **C** (n=3-5 per group). **E** Changes in blood glucose level measured after injection of insulin on mice and quantification of area under curve (n=3 per group). **F** Plasma insulin level in Rab7-CKO mice and wild type mice (n=3 per group). The Student's t-test was used to analyze the differences between 2 groups and the data are expressed as mean ± SD, ns: no significance and *p < 0.05, **p < 0.01 and ****p < 0.0001.

***Figure S8***


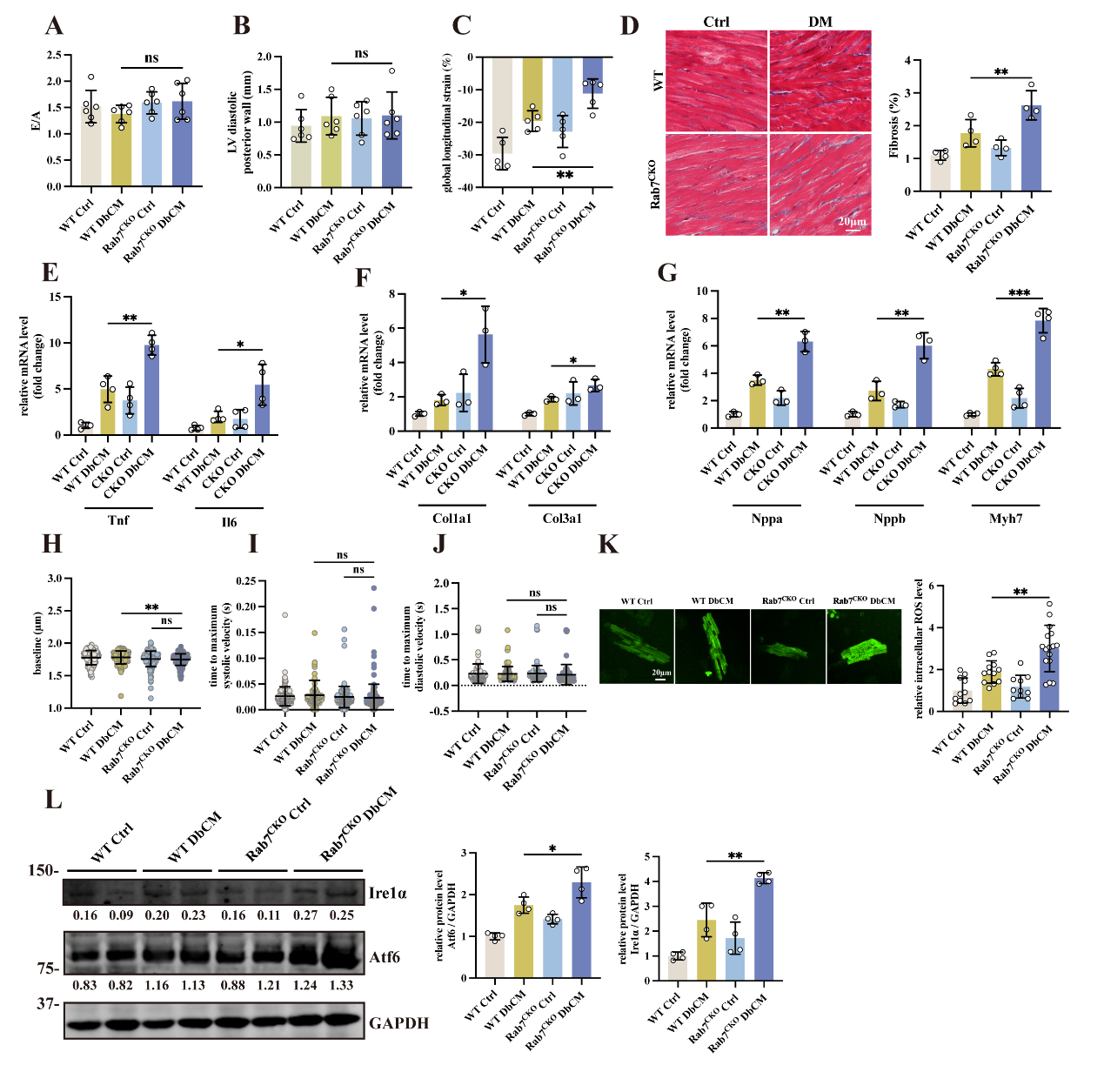


Figure S8. Rab7-CKO mice present severe lipid toxicity. **A** Evaluation of E/A waves ratio (n=6 per group). **B** Analysis on thickness of left ventricular diastolic posterior wall (LVPW:d) (n=6 per group). **C** Evaluation of global longitudinal strain (GLS) (n=5 per group). **D** Representative micrographs of Masson’s trichrome staining in heart sections of mice and quantification on fibrotic areas from Masson’s trichrome staining (n=4 per group). **E** Relative mRNA expression of Tnf and Il6 in DbCM mice hearts (n=3 per group). **F** Relative mRNA expression of Col1a1 and Col3a1 in DbCM mice hearts (n=3-4 per group). **G** Relative mRNA expression of Nppa, Nppb and Myh7 in DbCM mice hearts (n=3-4 per group). **H** Evaluation of baseline sarcomere length in Langendorff-isolated adult mouse cardiomyocytes (AMCMs) (at least 90 cells from 3 individual mice per group). **I** Evaluation of time to maximum systolic velocity in Langendorff-isolated adult mouse cardiomyocytes (AMCMs) (at least 90 cells from 3 individual mice per group). **J** Evaluation of time to maximum diastolic velocity in Langendorff-isolated adult mouse cardiomyocytes (AMCMs) (at least 90 cells from 3 individual mice per group). **K** Representative micrograph and quantitative analysis of intracellular reactive oxygen species (ROS) in Langendroff AMVMs (at least n=10 per group). **L** Representative blots, relative intensity and quantitative analysis of immunoblots analysis of Ire1α and Atf6 in Langendroff AMVMs (n=4 per group). The Student's t-test was used to analyze the differences between 2 groups and the data are expressed as mean ± SD, ns: no significance, *p < 0.05, **p < 0.01. ***p < 0.001.

***Figure S9***


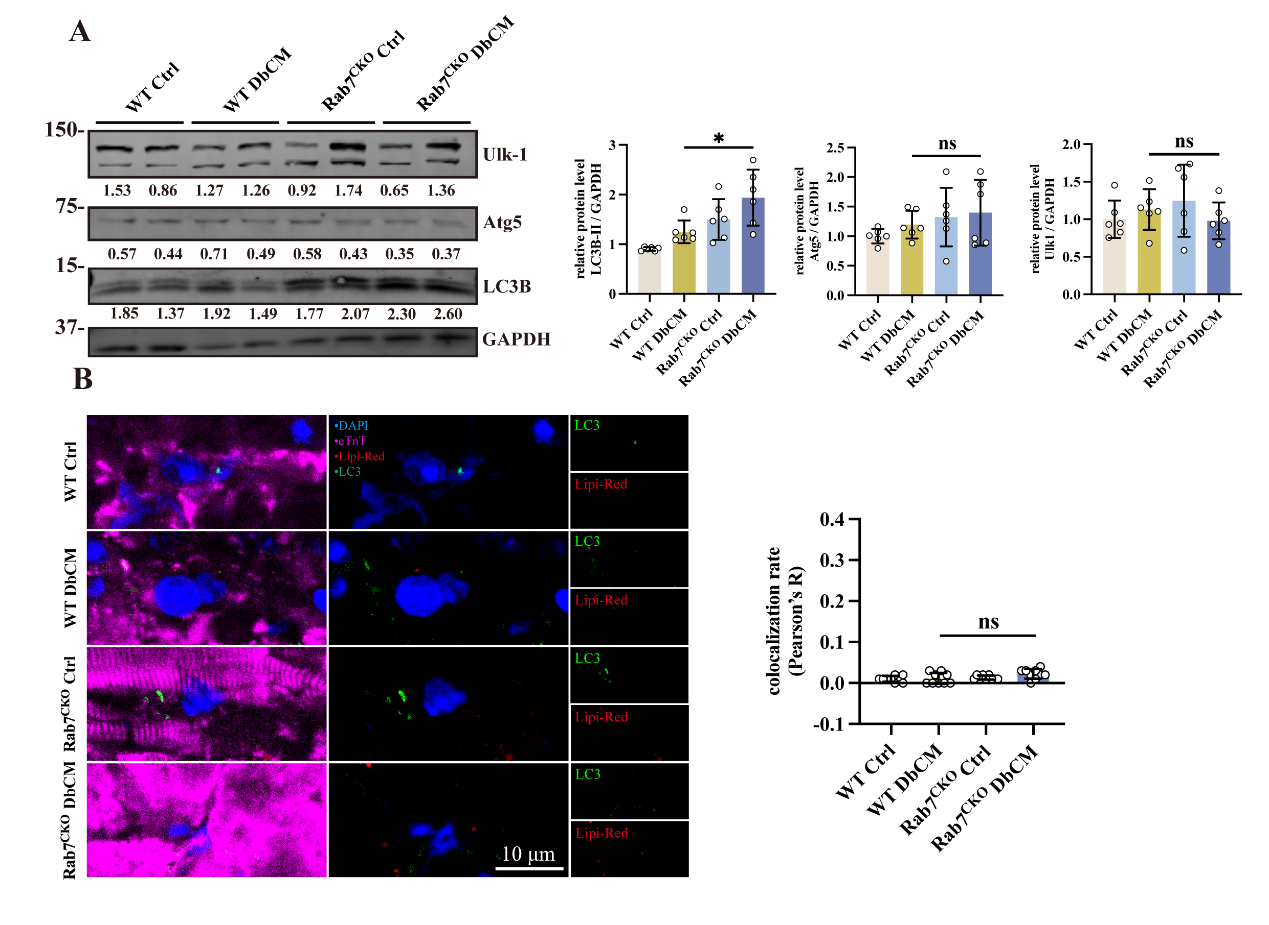


Figure S9. Loss of macroautophagic compensation in Rab7-CKO mice. **A** Representative blots, relative intensity and quantitative analysis of immunoblots analysis of Ulk1, Atg5 and LC3B in in Langendroff AMVMs (n=5-6 per group). **B** Representative micrographs of immunofluorescence double-staining of LC3 to label autophagosome and Lipi-Red (Red) to label LDs and quantification of Lipi-Red colocalizing LC3, results were presented as Pearson’ R (n=4-6 per group). The cell nuclei were stained with DAPI (blue) and cardiomyocytes were stained with cardiac troponin T (cTnT, magenta). The Student's t-test was used to analyze the differences between 2 groups and the data are expressed as mean ± SD, ns: no significance and *p < 0.05.

***Figure S10***


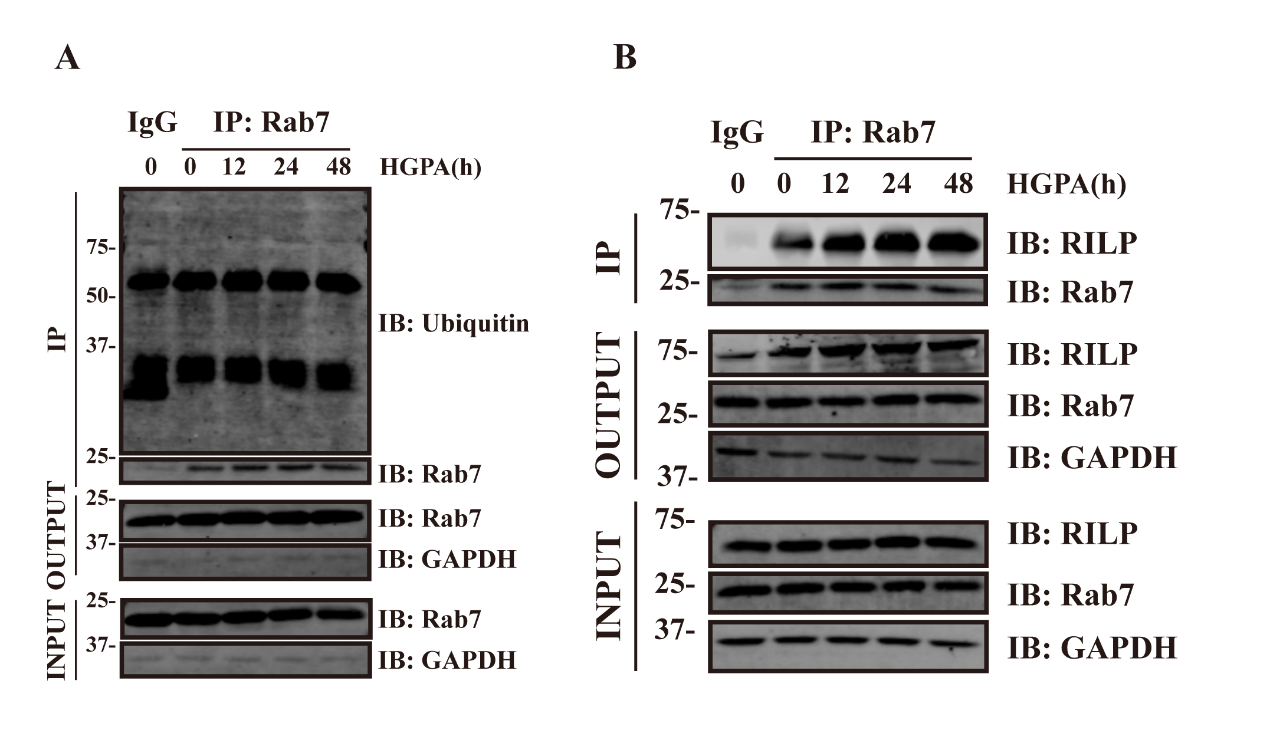


Figure S10. Rilp recruitment by Rab7 peaks after long-term HGPA stimulation. **A** Representative blot of immunoprecipitated Rab7 incubated with anti-Ubiquitin antibody after different time course of HGPA treatment. **B** Representative blot of Rab7 immunoprecipitated Rilp after different time course of HGPA treatment.

***Figure S11***


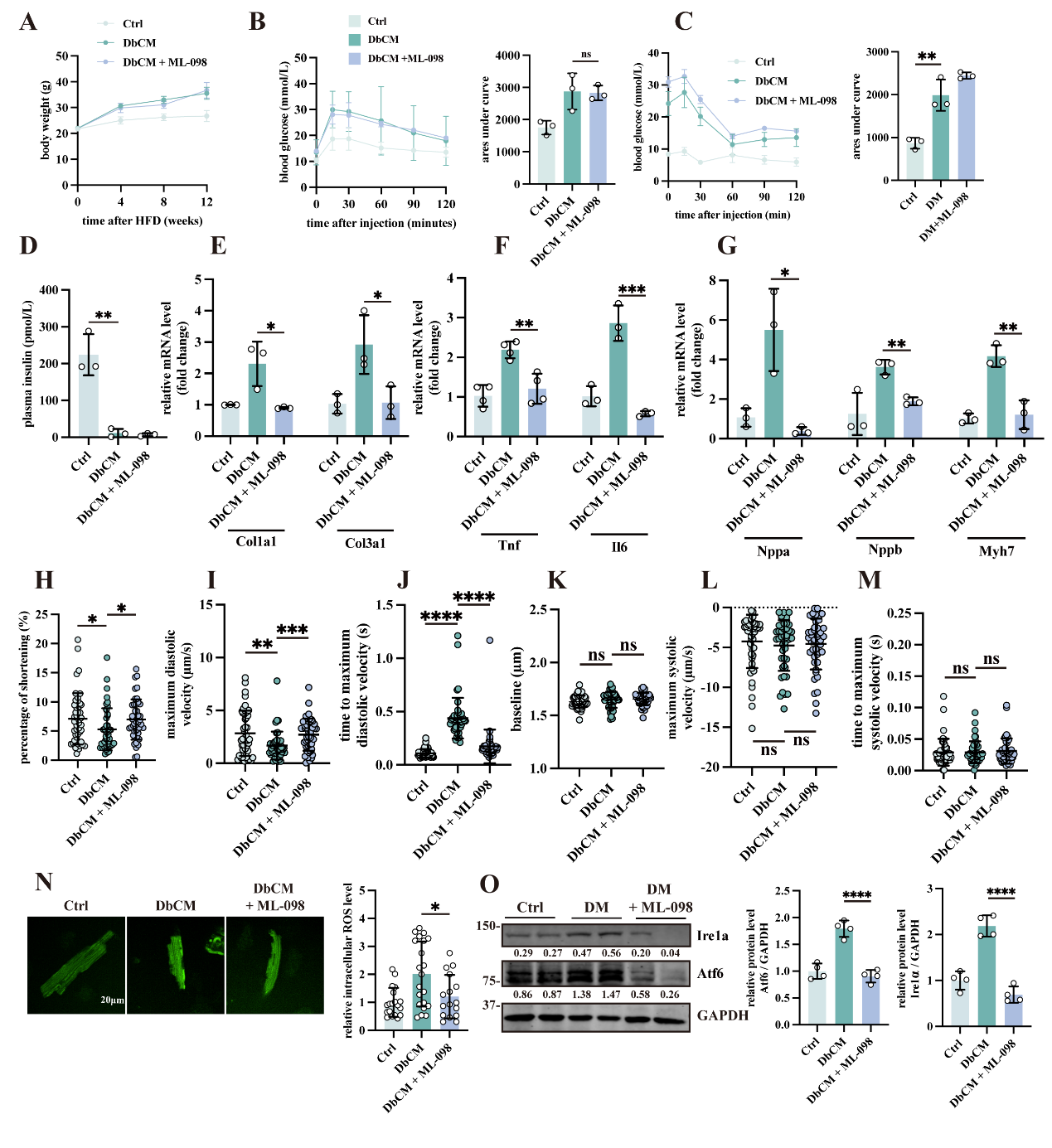


Figure S11. Activating Rab7 by ML-098 improves cardiac function in DbCM mice. **A** Recorded body weight of DbCM mice (n=3 per group). **B** Changes in blood glucose level measured after injection of α-d-glucose on mice and quantification of areas under curve (n=3 per group). **C** Changes in blood glucose level measured after injection of insulin on mice and quantification of areas under curve (n=3 per group). **D** Plasma insulin level in DbCM mice (n=3 per group). **E** Relative mRNA expression of Col1a1 and Col3a1 in DbCM mice hearts (n=3 per group). **E** Relative mRNA expression of Tnf and Il6 in DbCM mice hearts (n=3-4 per group). **G** Relative mRNA expression of Nppa, Nppb and Myh7 in DbCM mice hearts (n=3 per group). **H** Evaluation on percentage of shortening in Langendorff-isolated AMVMs (at least 45 cells from 3 individual mice per group). **I** Evaluation on maximum diastolic velocity in Langendorff-isolated AMVMs (at least 45 cells from 3 individual mice per group). **J** Evaluation of time to maximum diastolic velocity in Langendorff-isolated AMVMs. (at least 45 cells from 3 individual mice per group). **K** Evaluation of baseline sarcomere length in Langendorff-isolated AMVMs. (at least 45 cells from 3 individual mice per group). **L** Evaluation on maximum systolic velocity in Langendorff-isolated AMVMs (at least 45 cells from 3 individual mice per group). **M** Evaluation of time to maximum systolic velocity in Langendorff-isolated AMVMs (at least 45 cells from 3 individual mice per group). **N** Representative micrograph and quantitative analysis of intracellular reactive oxygen species (ROS) in Langendroff AMVMs (at least n=16 per group). **O** Representative blots, relative intensity and quantitative analysis of immunoblots analysis of Ire1α and Atf6 in Langendroff AMVMs (n=4 per group). The Student's t-test was used to analyze the differences between 2 groups and the data are expressed as mean ± SD, ns: no significance, *p < 0.05, **p < 0.01, ***p < 0.001 and ****p < 0.0001.

***Figure S12***


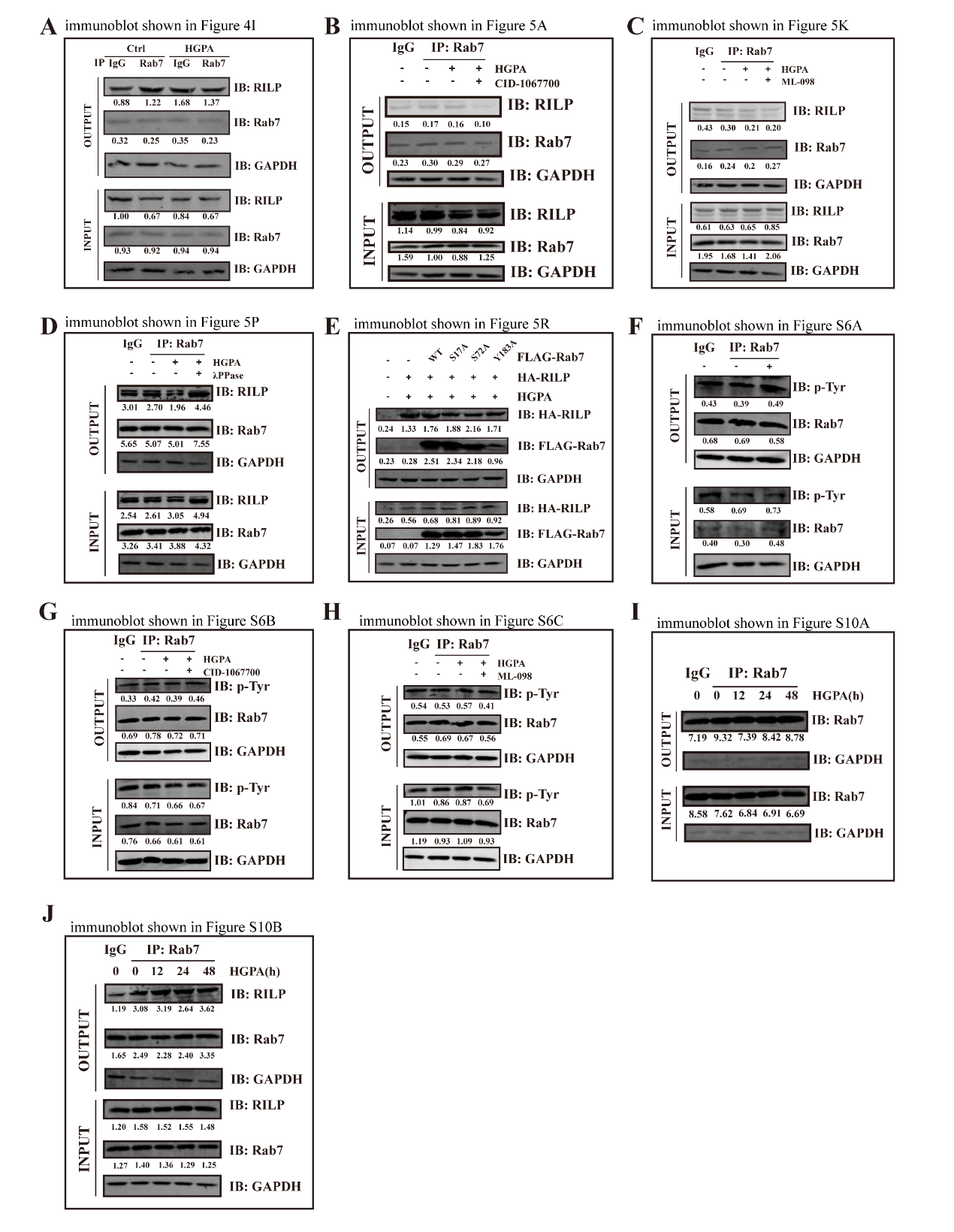


Figure S12. Quantification of relative intensity on immunoprecipitation results. **A** Quantification of relative intensity to GAPDH in Figure 4I. **B** Quantification of relative intensity to GAPDH in Figure 5A. **C** Quantification of relative intensity to GAPDH in Figure 5K. **D** Quantification of relative intensity to GAPDH in Figure 5P. **E** Quantification of relative intensity to GAPDH in Figure 5R. **F** Quantification of relative intensity to GAPDH in Figure S6A. **G** Quantification of relative intensity to GAPDH in Figure S6B. **H** Quantification of relative intensity to GAPDH in Figure S6C. **I** Quantification of relative intensity to GAPDH in Figure S10A. **J** Quantification of relative intensity to GAPDH in Figure S10B.
